# Homoharringtonine suppresses acute myeloid leukemia progression by orchestrating EWSR1 phase separation in an m^6^A‐YTHDF2‐dependent mechanism

**DOI:** 10.1002/imt2.70089

**Published:** 2025-10-30

**Authors:** Ting‐Ting Liu, Li‐Ting Chen, Xu‐Ying Pei, Shao‐Nan Hu, Fang‐Fang Zhuo, Ze‐Kun Chen, Yang Liu, Jing‐Kang Wang, Ji‐Chao Zhang, Qi Cao, Ling Li, Jing Wang, Tian‐Tian Wei, Bo Han, Peng‐Fei Tu, Xiang‐Yu Zhao, Ruidong Xue, Ke‐Wu Zeng

**Affiliations:** ^1^ State Key Laboratory of Natural and Biomimetic Drugs, School of Pharmaceutical Sciences Peking University Beijing China; ^2^ Institutes of Biomedical Sciences, School of Life Sciences Inner Mongolia University Hohhot China; ^3^ Yunnan Baiyao International Medical Research Center, International Cancer Institute and State Key Laboratory of Molecular Oncology, MOE Frontiers Science Center for Cancer Integrative Omics, School of Basic Medical Sciences Peking University Beijing China; ^4^ Peking University People's Hospital, Peking University Institute of Hematology, National Clinical Research Center for Hematologic Disease, Beijing Key Laboratory of Cell and Gene Therapy for Hematologic Malignancies, Peking University Beijing China; ^5^ College of Pharmacy Inner Mongolia Medical University Hohhot China; ^6^ Department of Integration of Chinese and Western Medicine, School of Basic Medical Sciences Peking University Beijing China; ^7^ Center of Basic Medical Research, Institute of Medical Innovation and Research Peking University Third Hospital Beijing China; ^8^ School of Pharmacy/Key Laboratory of Xinjiang Phytomedicine Resource and Utilization Shihezi University Shihezi China; ^9^ Translational Cancer Research Center Peking University First Hospital Beijing China

**Keywords:** acute myeloid leukemia, EWSR1, homoharringtonine, N^6^‐methyladenosine, phase separation, YTHDF2

## Abstract

Homoharringtonine (HHT) is widely used in combination regimens for acute myeloid leukemia (AML), yet its direct cellular targets remain undefined, limiting precision application. Here, we identified EWS RNA‐binding protein 1 (EWSR1) as the primary target of HHT through chemical proteomics and biophysical validation. HHT bound the RNA recognition motif of EWSR1 with micromolar affinity, inducing an allosteric conformational switch that promoted oligomerization and liquid–liquid phase separation (LLPS). EWSR1 condensates selectively recruited the N^6^‐methyladenosine (m^6^A) reader YTHDF2, forming cytoplasmic hubs where HHT disrupted YTHDF2–mRNA interactions. This sequestration attenuated m^6^A‐mediated RNA decay, stabilizing key transcripts such as *TNFRSF1B* and *HMOX1*, and thereby impairing AML cell proliferation. Integrated transcriptomics and single‐cell RNA‐seq analyses revealed that *EWSR1* was markedly upregulated in AML, particularly in hematopoietic progenitor and myeloid subpopulations, and high *EWSR1* expression correlated with poor prognosis and enhanced HHT sensitivity. In vivo, the anti‐leukemic efficacy of HHT was significantly diminished upon EWSR1 knockdown, demonstrating that EWSR1 was required for therapeutic response. Collectively, these findings uncover a phase separation‐centric mechanism by which HHT exerts anti‐AML activity, establish the EWSR1–YTHDF2–m^6^A *axis* as a critical regulator of leukemia progression, and position EWSR1 as both a functional target and a predictive biomarker for optimizing HHT‐based therapies.

## Highlights

- EWSR1 is identified as the direct cellular target of homoharringtonine (HHT) in acute myeloid leukemia (AML) through integrative chemical proteomics.
- HHT binding induces an allosteric conformational switch in EWSR1, promoting phase separation and sequestration of the m^6^A reader YTHDF2.
- EWSR1 functions as both a therapeutic effector of HHT and a predictive biomarker for treatment responsiveness in AML.

## Graphical abstract

Homoharringtonine (HHT), a clinically approved anti‐leukemic drug, directly binds the RNA‐binding protein EWSR1. HHT induces an allosteric conformational switch in the RNA recognition motif of EWSR1, driving self‐association and liquid–liquid phase separation (LLPS). The resulting condensates act as regulatory hubs that sequester the m^6^A reader YTHDF2, thereby preventing recognition and decay of m^6^A‐modified transcripts. Stabilization of apoptosis‐associated RNAs such as *TNFRSF1B* and *HMOX1* disrupts leukemic cell homeostasis and suppresses AML proliferation. These findings identify EWSR1 as the direct effector of HHT and a predictive biomarker for therapeutic responsiveness in AML.

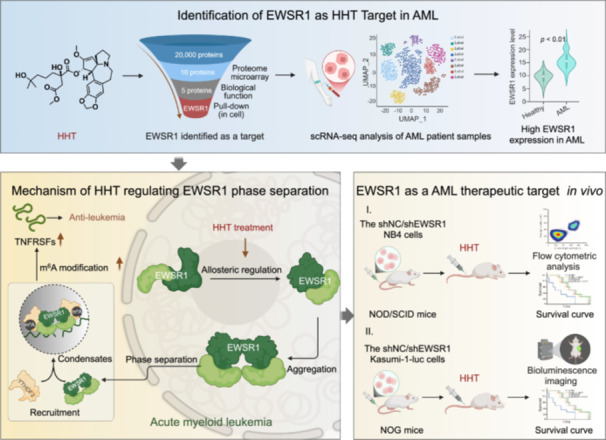

## INTRODUCTION

The primary treatment for acute myeloid leukemia (AML), particularly acute promyelocytic leukemia (APL), in clinical practice involves a combination therapy of arsenic trioxide and all‐trans retinoic acid. However, in most cases, this strategy should be used in conjunction with chemotherapy drugs for collaborative treatment. Homoharringtonine (HHT) was initially an agent approved by the Food and Drug Administration (FDA) for the treatment of chronic myeloid leukemia (CML) [1, 2]. Interestingly, HHT has been increasingly holding significant potential for the clinical treatment of AML in recent years [3]. In particular, the combination therapy of arsenic agent and all‐trans retinoic acid, when administered simultaneously with HHT, demonstrates potent synergistic effects, revealing a distinct anti‐leukemia mechanism [4, 5, 6]. However, the potential targets of HHT in the treatment of AML remain largely unexplored. Therefore, by assessing the expression levels of the target genes associated with HHT, we can identify which subtypes of AML patients may exhibit increased sensitivity to HHT therapy.

The EWS RNA‐binding protein 1 (EWSR1) is widely present across eukaryotic organisms and plays a pivotal role in diverse cellular mechanisms, including epigenetic processes associated with gene expression, RNA processing, and cellular signal transduction [7, 8]. There is a robust correlation between upregulated expression of EWSR1 and oncogenic transformations, indicating its potential role in the development of various cancers [9]. Specifically, recent investigations have unveiled a high prevalence of *EWSR1*‐associated chimeric fusion genes among AML patients [10], establishing a significant association between EWSR1 and the onset of AML. Moreover, multiple studies underscore the functional significance of EWSR1 in diverse cellular processes associated with AML progression, potentially involving pathways such as p53/p21 and JAK2 [11]. However, the molecular mechanisms that trigger AML progression by EWSR1 remain incompletely elucidated.

Liquid–liquid phase separation (LLPS) is thought to be a key process driving the formation of membraneless organelles. The EWSR1 consistently undergoes LLPS, exhibiting enhanced liquid‐like relaxation and diffusion characteristics. For instance, EWSR1 is recognized as a noteworthy contributor to the disruption of stress granule formation, which plays a vital role as membraneless compartments in cellular stress response mechanisms [12]. Moreover, the fusion protein of EWSR1 and FUS facilitates abnormal phase separation occurrences, leading to the erroneous recruitment of chromatin‐remodeling factors that trigger transcriptional events [13]. The EWS (also known as EWSR1)‐FLI1 fusion protein undergo phase separation at target binding sites, recruiting RNA polymerase II and enhancing gene transcription [14]. Therefore, the regulation of phase separation involving EWSR1 could potentially serve as an effective therapeutic approach against the progression of AML.

N^6^‐methyladenosine (m^6^A) is the predominant RNA modification that plays a crucial role in regulating the stability and translation of mRNA [15, 16, 17]. The presence of multiple m^6^A residues in mRNAs has been shown to significantly enhance phase separation [18]. Notably, the YTH domain family proteins (YTHDF1‐3) are key m^6^A reader proteins and have emerged as pivotal contributors to phase separation. For instance, the role of YTHDFs in phase separation is demonstrated by the formation of mRNA‐YTHDF complexes, which then partition into distinct endogenous compartments characterized by LLPS, such as P‐bodies, RNA granules, and stress granules. Meanwhile, YTHDF1 is demonstrated to drive the progression of AML [19]. YTHDF2 is overexpressed in various AML subtypes, and it is indispensable in initiating and perpetuating the disease [20]. Further, YTHDF2 is regulated by the AML1/ETO‐HIF1α loop and facilitates cellular proliferation by controlling global m^6^A methylation in AML [21]. Hence, the cumulative evidence emphasizes the central role of YTHDFs in AML development.

In this study, we identified EWSR1 as a specific cellular target of HHT in AML cells by synthesizing a biotin‐labeled molecule probe. Moreover, we observed that HHT directly binds to the RNA recognition motif of EWSR1 to obviously promote its phase separation. Surprisingly, our findings demonstrate that EWSR1 droplets play a specific role in facilitating the recruitment of YTHDF2, resulting in the weaken of m^6^A RNA decay targeting key factors involved in AML cell proliferation including TNF receptor superfamily members. Furthermore, our in vivo experiments confirmed that the knockdown of EWSR1 significantly mitigated the anti‐leukemic effect of HHT. Therefore, the involvement of EWSR1 in the regulation of YTHDF2 function in AML has significant clinical implications.

Overall, this study sheds light on a unique molecular mechanism involving EWSR1 phase separation in AML progression. Moreover, the identification of HHT as a small molecule targeting EWSR1 offers significant potential for developing effective therapies for AML patients with high EWSR1 expression levels.

## RESULTS

### EWSR1 is identified as a cellular target of HHT in AML

**Figure 1:**
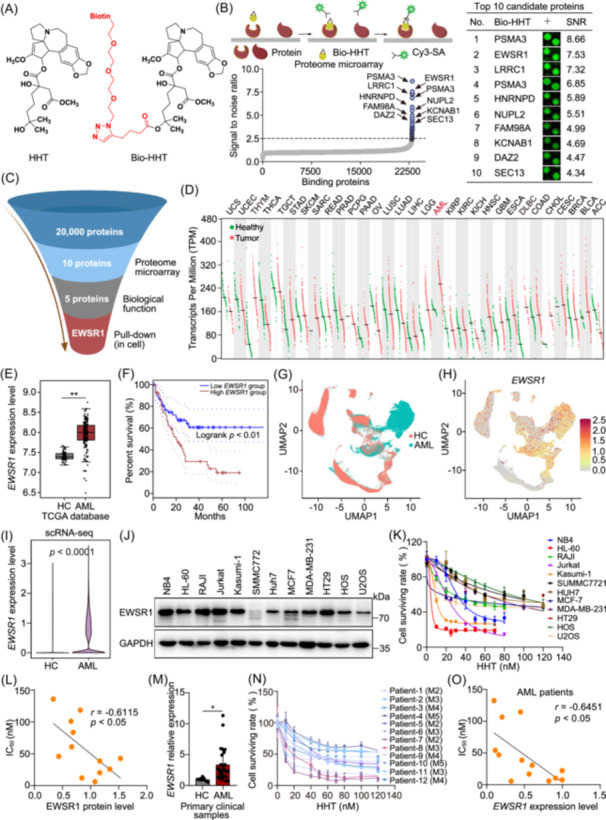
EWSR1 is identified as a cellular target of homoharringtonine (HHT). (A) The chemical structures of HHT and bio‐HHT analogs. (B) Identification of HHT target proteins using human proteome microarray. (C) Brief process for screening candidate target proteins of HHT. (D) *EWSR1* expression profile across 31 cancer types compared with normal tissues from the GEPIA database. The *x*‐axis lists cancer types by their TCGA abbreviations, with full names provided below: UCS (Uterine Carcinosarcoma), UCEC (Uterine Corpus Endometrial Carcinoma), THYM (Thymoma), THCA (Thyroid Carcinoma), TGCT (Testicular Germ Cell Tumors), STAD (Stomach Adenocarcinoma), SKCM (Skin Cutaneous Melanoma), SARC (Sarcoma), READ (Rectum Adenocarcinoma), PRAD (Prostate Adenocarcinoma), PCPG (Pheochromocytoma and Paraganglioma), PAAD (Pancreatic Adenocarcinoma), OV (Ovarian Serous Cystadenocarcinoma), LUSC (Lung Squamous Cell Carcinoma), LUAD (Lung Adenocarcinoma), LIHC (Liver Hepatocellular Carcinoma), LGG (Low‐Grade Glioma), AML (Acute Myeloid Leukemia), KIRP (Kidney Renal Papillary Cell Carcinoma), KIRC (Kidney Renal Clear Cell Carcinoma), KICH (Kidney Chromophobe), HNSC (Head and Neck Squamous Cell Carcinoma), GBM (Glioblastoma Multiforme), ESCA (Esophageal Carcinoma), DLBC (Diffuse Large B‐Cell Lymphoma), COAD (Colon Adenocarcinoma), CHOL (Cholangiocarcinoma), CESC (Cervical Squamous Cell Carcinoma and Endocervical Adenocarcinoma), BRCA (Breast Invasive Carcinoma), BLCA (Bladder Urothelial Carcinoma), ACC (Adrenocortical Carcinoma). (E) Box plots showing the expression profile of *EWSR1* in healthy tissues and acute myeloid leukemia (AML) from the TCGA database. num (Healthy) = 70, num (AML) = 173. **p < 0.01. (F) Kaplan‐Meier survival curves showing the association between *EWSR1* expression and overall survival in AML patients. Survival differences were evaluated using the log‐rank test. (G) UMAP of single‐cell transcriptomic data from healthy controls (HC) and AML individuals. (H) UMAP visualization of *EWSR1* expression in the HC and AML cells. Each dot represents a single cell, with color intensity reflecting normalized *EWSR1* expression levels. (I) Violin plot showing single‐cell *EWSR1* expression levels in HC and AML patients, based on normalized values (log_2_ scale). (J) Immunoblotting analysis of EWSR1 of a variety of leukemia cell lines and solid tumor cell lines. (K) Evaluation of antitumor effects of HHT on various leukemia and solid tumor cell lines. (L) Correlation between EWSR1 expression and HHT IC_50_ in the specified tumor cell lines. The Pearson correlation coefficient (*r*) is indicated. (M) Quantitative PCR analysis of *EWSR1* expression in AML patients (*n* = 26) and healthy controls (*n* = 11). *EWSR1* expression was significantly higher in AML samples (*p < 0.05, unpaired two‐tailed *t*‐test). (N) Concentration‐response curves showing the cell survival rates of primary AML cells from individual patients (*n* = 12) treated with increasing concentrations of HHT for 48 h. The cell survival was measured using CCK‐8 assay. Each line represents cells from a different patient (Patient‐1 to Patient‐12), with the corresponding French‐American‐British (FAB) subtype indicated in parentheses (M2−M5). (O) Correlation analysis between *EWSR1* expression levels and HHT sensitivity (IC_50_ values) in AML patients.

To elucidate the direct binding protein of homoharringtonine (HHT), we utilized a biotin‐labeled HHT probe (bio‐HHT) (Figure 1A, Figure S12) to screen a human proteome microarray containing over 20,000 recombinant proteins. We incubated bio‐HHT with proteome microarray, and the proteins that showed binding affinity for HHT were subsequently labeled using Cy3‐conjugated streptavidin (Cy3‐SA). Signal intensities were quantified as signal‐to‐noise ratio (SNR). This analysis identified 42 initial candidate proteins, which exhibited high confidence binding (SNR > 3). Based on the established positive correlation between SNR values and binding specificity in microarray experiments, we focused on the top 10 proteins with the highest SNR values (Figure 1B). From this subset, we prioritized five candidates (PSMA3, EWSR1, LRRC1, HNRNPD, and FAM98A) based on their SNR rankings, established roles in cancer pathways (e.g., proteasome function, RNA processing, and transcriptional regulation) [22, 23, 24, 25, 26]. HuProt^TM^ microarray uses purified recombinant proteins to assess binding affinity, but cellular pull‐down assays are more capable of capturing target proteins in the complex physiological environment of leukemia cells. Subsequent pull‐down assays, combined with Stable Isotope Labeling by Amino Acids in Cell Culture (SILAC), a quantitative global proteomics method, indicated that EWSR1 exhibited the most significant enrichment among all candidate targets. As shown in Figure 1C, Figure S3, the EWSR1 with high light/heavy ratio was identified as the crucial candidate target protein for HHT. While PSMA3, LRRC1, HNRNPD, and FAM98A demonstrated strong binding to HHT in vitro using purified proteins, their weaker engagement in leukemia cellular models highlights fundamental differences between simplified biochemical systems and complex physiological environments. To further elucidate the biological significance of *EWSR1* in AML, the relevant clinical data from the TCGA (The Cancer Genome Atlas) database were thoroughly analyzed. Transcriptomics studies in the database revealed a significant upregulation of *EWSR1* mRNA expression across various malignancies, particularly in AML tissues, when compared to normal tissues (Figure 1D,E). Additionally, the prognostic significance of *EWSR1* in patients diagnosed with AML was evaluated utilizing the Kaplan–Meier (KM) method. Our findings indicated a remarkable association between increased expression of *EWSR1* and poor overall survival in AML patients (Figure 1F), thus suggesting a positive linkage between *EWSR1* and the progression of AML.

To further investigate the correlation between *EWSR1* and clinical AML progression, we integrated publicly available single‐cell RNA sequencing (scRNA‐seq) datasets from healthy individuals and AML patients (GSE120221 and GSE241989), resulting in a total of 178,532 high‐quality single‐cell data. UMAP visualization colored by 19 AML patients and 20 healthy controls (HCs) showed the obvious transcriptome changes of bone marrow in AML (Figure 1G). The UMAP visualization and violin plot showed significantly higher expression of *EWSR1* in AML patients compared to healthy individuals (Figure 1H,I, Figure S4A). Given the similar differentiation hierarchy in bone marrow between AML and healthy controls, all cells were categorized into four lineages based on canonical hematopoietic lineage markers, including hematopoietic stem progenitor cells (HSPC) characterized by *CD34*, myeloid cells by *LYZ*, *CD14*, and *FCGR3B*, erythroid cells by *HBA*, and lymphoid cells by *CD3D* and *CD79A* (Figure S4B). In AML patients, HSPC and myeloid cells accounted for approximately 50%–90% of each sample, whereas lymphoid cells and erythroid cells predominated in healthy individuals (Figure S4C). Based on unsupervised clustering, these lineages were further divided into 21 subpopulations. Single‐cell analysis indicated that *EWSR1* was more highly expressed in AML patients, particularly in HSPC subpopulations including CD34^+^ HSC, cycling CD34^+^ HSC, multipotent progenitors (MPP), cycling MPP, and mast cell progenitor (MCP), as well as in myeloid subpopulations such as granulocyte monocyte progenitor (GMP), pro‐neutrophil, CD14+ monocyte, CD16+ monocyte, and dendritic cells (DCs) (Figure S4D–F). Taken together, *EWSR1* ^high^ HSPC and *EWSR1* ^high^ myeloid dominate in AML bone marrows, indicating *EWSR1* was a crucial therapeutic target for AML intervention.

Next, western blot analysis was conducted to evaluate EWSR1 protein expression across various tumor cell lines. Our findings revealed that EWSR1 expression was significantly higher in AML leukemia cells compared to solid tumor cells (Figure 1J, Figure S5A). HHT demonstrated broad‐spectrum inhibitory effects on multiple tumor cell lines in vitro, especially on AML cells such as HL‐60, NB4, and Kasumi‐1 (Figure 1K). Meanwhile, a negative correlation was identified between EWSR1 protein levels and HHT IC_50_ values, as indicated by the Pearson correlation coefficient (Figure 1L). Additionally, we evaluated the efficacy of HHT in a panel of AML cell lines, including Kasumi‐1, NB4, HL‐60, U937, THP‐1, OCI‐AML2, and HEL (Figure S5B). These models represent major FAB subtypes (M2‐M6) and encompass diverse genetic backgrounds. HHT exhibited potent anti‐leukemic activity across all tested cell lines, with IC_50_ values consistently in the low nanomolar range. Notably, HHT sensitivity was negatively correlated with *EWSR1* expression (Pearson *r* = −0.7972, *p* = 0.0318, Figure S5C), indicating that higher *EWSR1* levels are associated with increased HHT susceptibility. These findings further support a critical role for EWSR1 in mediating the anti‐leukemic effects of HHT in vitro. Additionally, evaluation of primary patient‐derived samples demonstrated a significant elevation of *EWSR1* expression in AML specimens relative to healthy controls (Figure 1M). Primary AML cells from 12 patients representing FAB subtypes M2‐M5 were treated with increasing concentrations of HHT, revealing potent anti‐leukemic effects with low nanomolar IC_50_ values (Figure 1N). Notably, *EWSR1* expression levels were negatively correlated with HHT IC_50_ values (Pearson *r* = −0.6451, *p* = 0.0173), indicating that higher *EWSR1* expression predicted greater HHT sensitivity in AML patients (Figure 1O). In summary, this significant correlation underscores a potential relationship between EWSR1 expression and the responsiveness of AML cells to HHT treatment.

### RNA recognition motif of EWSR1 mediates HHT binding

**Figure 2:**
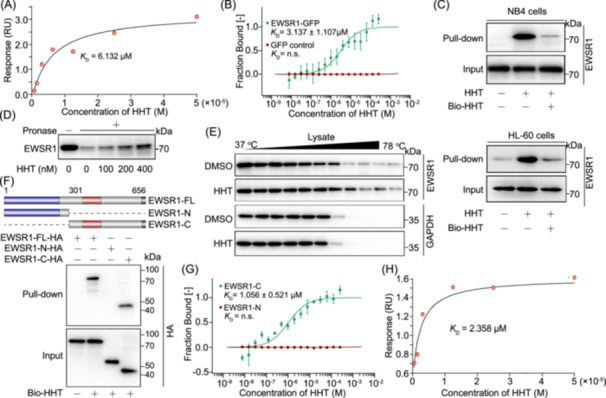
HHT binds the RNA recognition motif of EWSR1. (A) Surface plasmon resonance (SPR) assay showed the binding affinity of HHT with EWSR1. (B) HHT binding to EWSR1 was analyzed using microscale thermophoresis (MST), yielding a dissociation constant of 3.137 ± 1.107 μM, calculated from three separate experiments. (C) Pull‐down assay verified the engagement of HHT with EWSR1 in NB4 and HL60 cells. The EWSR1 immunoprecipitated with HHT beads was analyzed by western blot. (D) HHT enhanced EWSR1 resistance to proteases, which was analyzed by drug affinity responsive target stability (DARTS) assay. (E) HHT facilitated the resistance of EWSR1 to varying temperature gradients via cellular thermal shift assay (CETSA) analysis. (F) Pull‐down assay of HHT binding to the domain of EWSR1. (G) MST analysis revealing a direct binding of HHT with EWSR1‐C in lysates from HEK293T cells overexpressing GFP‐EWSR1‐C. (H) SPR analysis of HHT binding to EWSR1‐C recombinant protein.

To further confirm the direct interaction between HHT and EWSR1, we performed SPR analysis, which demonstrated a robust binding with a *K*_D_ value of 6.132 μM (Figure 2A). In parallel, we employed MST to validate this interaction (*K*_D_ value of 3.137 ± 1.107 μM) (Figure 2B). Moreover, pulldown experiments were performed to capture the EWSR1 protein in NB4 and HL‐60 cells using a biotin‐labeled HHT probe. The protein level of EWSR1 binding with HHT significantly increased in comparison to the control group, thereby confirming the interaction between HHT and EWSR1. The introduction of free HHT for competitive binding effectively reversed this interaction (Figure 2C). Additionally, we adopted drug affinity responsive target stability (DARTs) analysis to corroborate the interaction between HHT and EWSR1 (Figure 2D). Meanwhile, these findings were further validated through CETSA experiments, revealing a substantial binding affinity between HHT and EWSR1 (Figure 2E). To elucidate the specific domain of EWSR1 protein that interacts with HHT, we performed a division of the protein into distinct N‐terminal (amino acids 1–300) and C‐terminal (amino acids 301–656) regions (RNA recognition motif). Pull‐down experiments confirmed that HHT directly interacted with the C‐terminal of EWSR1 (Figure 2F, Figure S6). Subsequently, we successfully expressed the C‐terminal protein and performed both MST and SPR experiments. Remarkably, our findings consistently demonstrated a specific interaction between HHT and the EWSR1 C‐terminal region (Figure 2G,H). In summary, our findings provide compelling evidence for a direct interaction between HHT and the RNA recognition motif of EWSR1.

### HHT binds the RNA recognition motif of EWSR1 through hydrogen bonds

**Figure 3:**
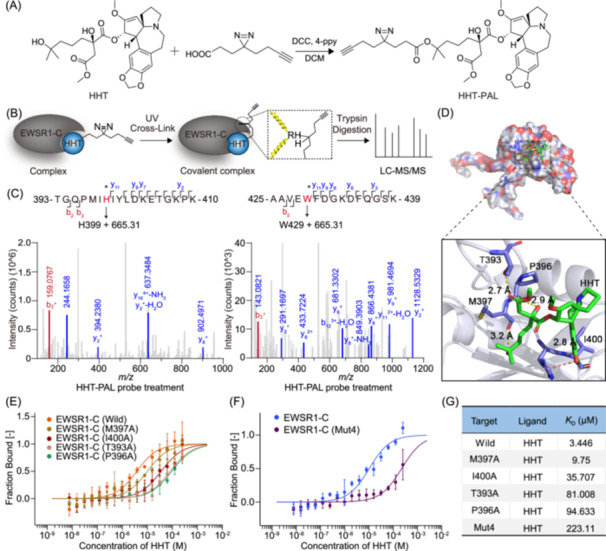
HHT interacts with the RNA recognition motif through hydrogen bonds. (A) The synthesis of HHT‐PAL probe. (B) Schematic representation of covalently modified peptide identified by HHT‐PAL probe in EWSR1‐C with click chemical probe. (C) Liquid chromatography‐tandem mass spectrometry (LC‐MS/MS) analysis of covalently modified peptide of HHT‐PAL probe binding to EWSR1‐C. (D) Molecular docking of HHT toward the structure of EWSR1 (353–453) protein (PDB: 2CPE). (E, F) MST analysis demonstrating the direct interaction between HHT and EWSR1‐C mutants, including single‐point mutations and multiple‐point mutation. (G) The binding constants of these EWSR1‐C mutants are provided in the table.

To further investigate the key amino acids involved in the interaction between HHT and EWSR1, we synthesized a photoaffinity labeling probe for HHT (HHT‐PAL) to investigate the potential modified peptides in the C‐terminal region of EWSR1, particularly the RNA recognition motif (Figure 3A, Figure S7). As shown in Figure 3B, upon binding of EWSR1‐C with HHT‐PAL, a covalent crosslinking occurred via the diazirine moiety under UV light irradiation. The binding peptides were subsequently identified using liquid chromatography‐tandem mass spectrometry (LC‐MS/MS) analysis. The results revealed the presence of multiple peptide segments exhibiting a molecular weight increase of 665.3194, which corresponds to the HHT‐PAL probe (Figure 3C, Figure S8). Importantly, two specific peptide sequences, 393‐TGQPMIHIYLDKETGKPK‐410 and 425‐AAVEWFDGKDFQGSK‐439, were identified as potential binding peptides for the modification of HHT probe. Therefore, the precise site of crosslinking was identified to be within the RNA recognition motif (353–453) of EWSR1. We investigated the binding of HHT to the RNA recognition motif of EWSR1 (PDB code: 2CPE) using molecular docking (Figure 3D). The result showed that HHT formed hydrogen bonds with four residues (T393, P396, M397, and I400) in EWSR1. Additionally, the formation of a π–π interaction between HHT and the H399 residue was observed. To further investigate whether these amino acids are involved in mediating the interaction between EWSR1 and HHT, we constructed several single‐point and multiple‐point mutants of EWSR1‐C. The binding affinity of HHT to these EWSR1 mutants was assessed through MST and pull‐down experiments. As shown in Figure 3E–G, the EWSR1 mutants exhibited a weaker affinity compared to HHT, suggesting that the residues T393, P396, and I400 were essential for the interaction between HHT and RNA recognition motif. In the pull‐down assays, wild‐type (WT) EWSR1 exhibited a strong band, indicating robust binding to HHT. The M397A mutant, used as a noncritical residue control, displayed binding comparable to WT. In contrast, mutations at T393A, P396A, and I400A, which were predicted to be critical for the interaction, resulted in markedly reduced HHT binding, as evidenced by significantly weaker bands. The Mut4 construct similarly showed diminished binding (Figure S9). These findings provide direct experimental evidence that residues T393, P396, and I400 within the RRM domain of EWSR1 are essential for HHT binding, strongly supporting our computational predictions and MST data. Therefore, HHT may bind to EWSR1 through multiple hydrogen bonds, thereby mediating the downstream cell signaling pathways.

### HHT promotes the droplet formation of the EWSR1 protein

**Figure 4:**
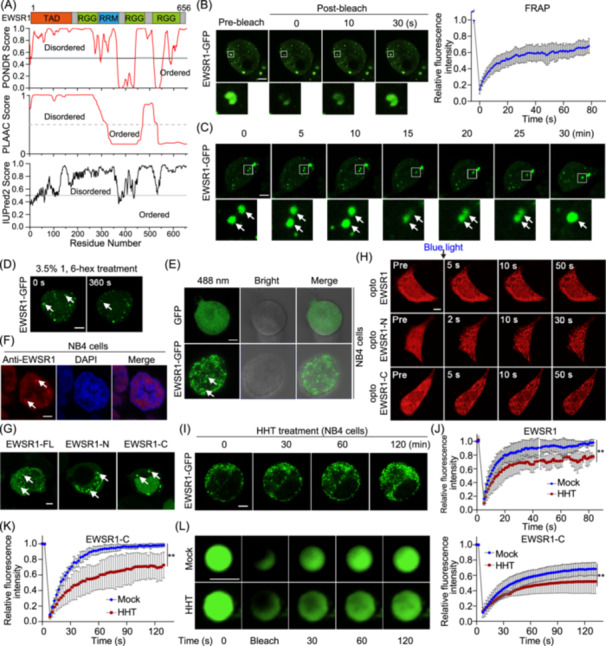
HHT promotes liquid–liquid phase separation of EWSR1 protein. (A) Predictions of disordered regions using predictor of natural disordered regions (PONDR), prion‐like amino acid composition (PLAAC), and prediction of intrinsically unstructured proteins (IUPred2) algorithms. A probability score of less than 0.5 indicates an ordered region, while above 0.5 indicates a disordered region. The schematic representation of the EWSR1 protein structure is provided above. (B) Captured on the left are representative micrographs, while on the right is the fluorescence recovery after photo bleaching (FRAP) quantification, illustrating EWSR1‐GFP condensates in vivo before and after photobleaching (white frame indicating bleach site) over an 80s period (*n* = 3 condensates). Scale bar: 10 μm. (C) Fusion of EWSR1‐GFP condensates in HEK293T cells. Scale bar: 10 μm. (D) Living imaging displaying HEK293T cells transfected with EWSR1‐GFP and either treated with 3.5% 1,6‐hexanediol or left untreated. Scale bar: 5 μm. (E) The formation of EWSR1‐GFP condensates in NB4 cells. Scale bar: 2 μm. (F) Immunofluorescence (IF) of EWSR1 in NB4 cells. The nuclei were labeled with DAPI. Scale bar: 2 μm. (G) Images showing the localization of GFP‐tagged EWSR1 truncated proteins. (H) Representative images depicting the clustering of EWSR1 (FL), EWSR1‐N, and EWSR1‐C‐Cry2 under blue‐light stimulation at various time points in HEK293T cells. Cells were exposed to a 488 nm laser. Scale bar: 5 μm. (I) Representative micrographs showed a rapid increase in puncta formation in NB4 cells following HHT treatment. Scale bar: 2 μm. (J) Quantitative FRAP assay of EWSR1‐GFP with or without HHT in HEK293T cells. Data are represented as mean ± SD (*n* = 4). (K) Quantitative FRAP assay of EWSR1‐C‐GFP with or without HHT in HEK293T cells. Data are shown as mean ± SD (*n* = 5). (L) Representative images and quantitative analysis of FRAP assays of EWSR1‐C‐GFP in vitro, with or without HHT treatment. Data are presented as mean ± SD (*n* = 5). Scale bar: 5 μm. Statistical significance for (J–L) was determined by unpaired two‐tailed Student's *t*‐test (**p < 0.01).

Previous studies have indicated that EWSR1 tends to undergo LLPS and form protein droplets within cellular environments [27]. In this study, we assessed the impact of various structural domains of EWSR1 on its phase separation phenomenon via bioinformatics analysis. The investigation unveiled numerous intrinsically disordered regions within EWSR1, specifically an N‐terminal transcriptional activation domain and a C‐terminal RGG‐rich domain. These domains played a crucial role in promoting the LLPS of full‐length EWSR1 (Figure 4A). To validate these findings, we transfected HEK293T cells with a GFP‐tagged EWSR1 expression vector and then performed fluorescence recovery after photobleaching (FRAP) analysis on a defined region within an individual droplet. The focal points displayed pronounced recovery within 80 s following photobleaching, providing evidence of their dynamic and liquid‐like properties (Figure 4B). Meanwhile, these specks exhibited liquid‐like properties shown by droplet fusion (Figure 4C). The application of 1, 6‐hexanediol, a chemical recognized for its disruptive impact on liquid‐like condensates, exhibited a substantial decrease in the abundance of EWSR1 puncta (Figure 4D). Additionally, our findings confirmed the occurrence of EWSR1 phase separation in NB4 cells (Figure 4E,F).

To investigate which domain of EWSR1 contributes to phase separation, we designed a series of GFP‐labeled constructs and monitored their capability of driving puncta formation. The result showed that EWSR1‐N and EWSR1‐C formed obvious puncta resembling full‐length EWSR1 (Figure 4G). We further utilized the optoDroplets assay to investigate the underlying domain that drives EWSR1 phase separation. The optoDroplets system was established by genetically fusing Cry2, a light‐responsive protein that undergoes self‐association upon exposure to blue light (Figure S10A), with EWSR1, EWSR1‐N, and EWSR1‐C domains. This property of Cry2 serves as a “seed” for initiating condensate formation in intrinsically disordered regions (IDRs). Time‐lapse imaging demonstrated that the application of blue light resulted in robust formation of droplets composed of EWSR1‐N and EWSR1‐C (Figure 4H). Furthermore, phase‐contrast microscopy revealed the formation of EWSR1‐C protein droplets in vitro, which were further enhanced by HHT treatment (Figure S10B). The time‐lapse imaging revealed that HHT gradually facilitated the phase separation of EWSR1, resulting in increased condensates within 2 h in NB4 cells (Figure 4I, Figure S10C). Given that HHT may alter the phase separation behavior of EWSR1, we performed a FRAP assay. The results indicated that HHT significantly reduced the fluorescence recovery rate of both EWSR1 and EWSR1‐C droplets (Figure 4J,K). We speculate that HHT may affect the conformation of EWSR1‐C, thereby altering the physicochemical properties of LLPS. To this end, in vitro assays demonstrated that HHT promoted the formation of EWSR1‐C condensates and increased droplet density (*p* < 0.05). Moreover, HHT markedly prolonged the fluorescence recovery time of EWSR1‐C, suggesting that it may drive a liquid‐to‐solid transition of EWSR1‐C condensates in vitro (Figure 4L), thereby reducing their circularity and mobility. Thus, these results indicate the significant phase separation characteristic of EWSR1 in AML cells, which can be effectively regulated by HHT.

### HHT facilitates EWSR1 phase separation via an allosteric mechanism

**Figure 5:**
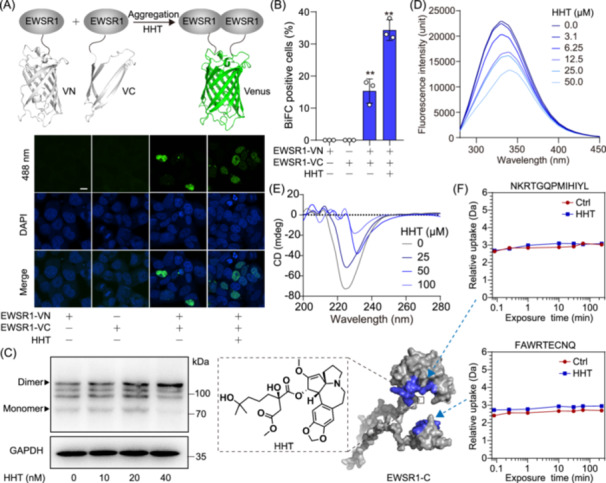
HHT facilitates EWSR1 phase separation via an allosteric regulation. (A) Schematic illustration depicting the binding of EWSR1 in a bimolecular fluorescence complementation (BiFC) assay. Representative confocal images displaying cells with positive BiFC signals. The HEK293T cells were transfected with *EWSR1*‐VN and *EWSR1*‐VC. The nuclei were stained with DAPI. Scale bar: 10 μm. (B) Quantitative analysis of BiFC positive cells. Bars represent mean ± SD; **p < 0.01. (C) Western blot images for EWSR1 oligomerization in NB4 treated with HHT at different concentrations. (D) Fluorescence spectroscopy analysis of conformational changes in EWSR1‐C induced by HHT. The fluorescence emission spectra of recombinant EWSR1‐C, obtained upon excitation at 280 nm, were measured with different concentrations of HHT. (E) Analysis of CD spectra for HHT‐mediated conformational changes in EWSR1‐C. (F) HHT allosterically regulated the conformation of EWSR1‐C through hydrogen‐deuterium exchange mass spectrometry (HDX‐MS) analysis.

To investigate the mechanism of EWSR1 phase separation, we constructed a Bimolecular Fluorescence Complementation (BiFC) reporter system [28, 29, 30]. Given the robust fluorescence properties exhibited by the venus protein, we divided its structure into slightly overlapping fragments, the N‐terminal (VN173) and C‐terminal (VC155) fragment. Subsequently, we independently integrated these two domains either at the C‐terminal region of EWSR1. Consequently, we achieved successful acquisition of two vectors: EWSR1‐VN and EWSR1‐VC. After co‐transfecting these two expression vectors, the direct interaction between EWSR1 variants leads to the physical association of VN and VC domains, resulting in the formation of a fully functional venus protein that visibly fluoresces. As illustrated in Figure 5A,B, the BiFC assays revealed the self‐interaction of EWSR1, indicating the potential formation of homodimers or oligomers. Furthermore, this preference is significantly increased by the presence of HHT, demonstrating that HHT targets the C‐terminal region of EWSR1 to effectively accelerate its self‐interaction. Additionally, the immunoblotting analysis revealed that the oligomerization of EWSR1 significantly increased by HHT in a concentration‐dependent manner (Figure 5C). We hypothesize that the self‐assembly mechanism could be facilitated by conformational alterations of the EWSR1‐C protein induced by HHT. Fluorescence analysis of tryptophan was conducted to explore the possible role of HHT in the EWSR1‐C conformation. HHT resulted in a noticeable reduction in the fluorescence intensity of EWSR1‐C, indicating its potential to induce conformational alterations in EWSR1‐C (Figure 5D). Next, circular dichroism (CD) spectroscopy was utilized to evaluate the secondary structure of EWSR1‐C. Upon subjecting EWSR1‐C to various concentrations of HHT, a gradual decline in molar ellipticity measurements at 222 nm was observed, indicating a direct relationship between the reduction in protein helicity and changes in concentration (Figure 5E). This shift may further enhance protein aggregation. Then, we utilized hydrogen‐deuterium exchange mass spectrometry (HDX‐MS) to investigate the allosteric mechanism. The results showed that HHT increased the HDX rates of two particular peptides (NKRTGQPMIHIYL, FAWRTECNQ) (Figure 5F). In particular, the NKRTGQPMIHIYL peptide was essential for RNA recognition and the binding of HHT. Taken together, these findings elucidate that HHT allosterically regulates EWSR1, thereby promoting protein oligomerization and facilitating the rapid formation of protein droplets.

### HHT enhances EWSR1–YTHDF2 interaction to suppress m^6^A recognition

**Figure 6:**
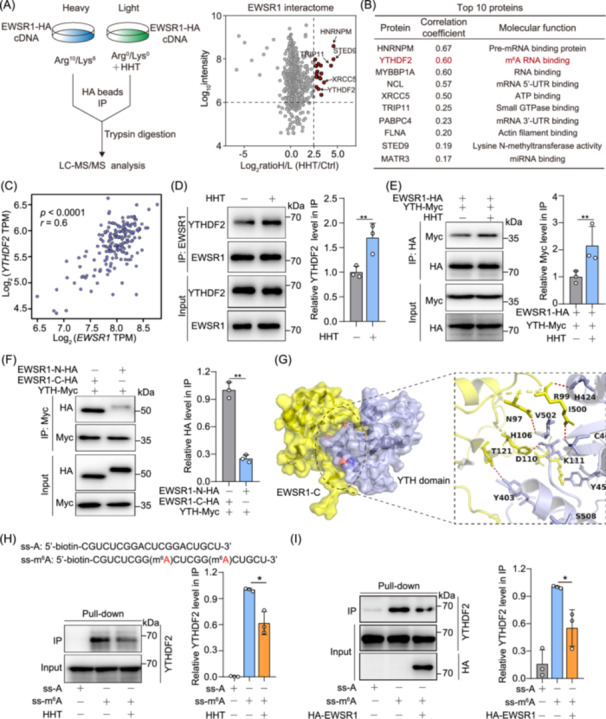
HHT strengthens EWSR1–YTHDF2 interaction, restricting m^6^A recognition. (A) Illustrative representation of a proteomic assay utilizing Stable Isotope Labeling by Amino Acids in Cell Culture (SILAC) (Left). The identification of proteins interacting with EWSR1 via a combination of co‐immunoprecipitation (Co‐IP) and mass spectrometry assays. Scatter plot of all proteins identified was showing on the right. (B) Top ten proteins interacting with EWSR1 are listed in the table. (C) Correlation analysis between *EWSR1* and *YTHDF2* mRNA expression from the AML patients. Pearson correlation coefficient (*r*) is 0.6. (D) The interaction between EWSR1 and YTHDF2 was assessed using a CO‐IP assay in NB4 cells. Quantification of YTHDF2 enrichment in IP fractions was measured using ImageJ software. (E) Mapping YTHDF2 regions binding to EWSR1. EWSR1‐HA and YTH^YHTDF2^‐myc were simultaneously transfected into HEK293T cells. Cell extracts were IP with anti‐HA antibodies. Right: Quantification of Myc levels in IP fractions (normalized to input Myc levels) was shown. (F) Mapping EWSR1 regions binding to YTH^YHTDF2^. HEK293T cells were cotransfected with indicated constructs of EWSR1 and YTH^YHTDF2^‐myc. Cell extracts underwent IP using anti‐Myc antibodies. Quantification of HA (EWSR1) levels in IP fractions (normalized to input HA levels) was presented. (G) Molecular docking of YTH domain (PDB: 4RDO) toward the structure of EWSR1‐C. (H) Pull‐down assay of YTHDF2 incubated with m^6^A RNA probe or unmethylated RNA probe under HHT treatment. Right: Quantification of YTHDF2 enrichment in IP fractions (normalized to input YTHDF2 levels) was shown. (I) Pull‐down analysis of YTHDF2 binding to m^6^A with EWSR1 overexpression. Right: Quantification of relative YTHDF2 enrichment in IP fractions (normalized to input YTHDF2 levels). Data represent mean ± SEM from three independent experiments (*p < 0.05, **p < 0.01).

To understand the proteins that can be recruited by EWSR1 droplets, we attempted to identify the protein profile within the droplets formed through phase separation. As shown in Figure 6A, we performed co‐immunoprecipitation (Co‐IP) in combination with SILAC‐based quantitative proteomics analysis. Then, we identified potential binding substrate proteins of EWSR1, such as HNRNPM and YTHDF2, which have previously been implicated in the formation of protein droplets (Figure 6B). Notably, we were particularly intrigued by YTHDF2 due to its fundamental involvement in protein phase separation and its strong correlation with tumor cell proliferation. Based on the analysis of TCGA datasets, we found that *YTHDF2* expression was positively correlated with *EWSR1* expression in AML patients (Figure 6C). Based on the scRNA‐seq datasets from healthy individuals and AML patients, we further explored the expression pattern of YTHDF2. UMAP by YTHDF2 expression level exhibiting a similar distribution pattern to that of *EWSR1* (Figure S11A). The expression of YTHDF2 was higher in AML compared to HCs, especially in HSPC and myeloid subpopulations (Figure S11B–E). Furthermore, a significant correlation between expression levels of *EWSR1* and *YTHDF2* was detected at the single‐cell level, showing strong co‐expression pattern of *EWSR1* and *YTHDF2* in AML patients (Figure S11F,G). Differential gene expression analysis further confirmed that both *EWSR1* and *YTHDF2* were significantly upregulated in AML patients compared to healthy individuals (Figure S11H). Taken together, these findings offer significant insights into the molecular mechanisms of AML, highlighting the potential of YTHDF2 as a key downstream effector of EWSR1 in driving AML progression.

To further explore the direct interaction between EWSR1 and YTHDF2, we conducted Co‐IP experiments. Our findings revealed a significant enhancement of the interaction between EWSR1 and YTHDF2 in both 293 T cells and NB4 cells when treated with HHT (Figure 6D, Figure S11I). Additionally, HHT enhanced the interaction between EWSR1 and the YTH domain of YTHDF2 (Figure 6E). We further investigated the interaction between the YTH domain of YTHDF2 and a specific domain of EWSR1. The results indicated that the C‐terminal region of EWSR1 interacted with the YTH domain, which specifically recognizes m^6^A‐modified RNA sites (Figure 6F). Molecular docking showed that the EWSR1‐C truncation specifically interacted with the YTH domain of YTHDF2 through a unique protein–protein interaction interface (Figure 6G). Based on the established connection between YTHDF2 and RNA m^6^A modification in cellular processes, we hypothesize that the EWSR1 phase separation may play a significant role in governing intracellular RNA stability by recruiting YTHDF2. HHT promoted the interaction between EWSR1 and YTHDF2, driving the formation of cytoplasmic condensates that restricted YTHDF2's ability to recognize m^6^A‐modified RNA. In vehicle‐treated cells, YTHDF2 displayed a diffuse cytoplasmic localization with broad m^6^A co‐localization. However, after HHT treatment, YTHDF2 was sequestered into EWSR1‐containing puncta, leading to reduced m^6^A recognition activity (Figure S11J). These results indicate that EWSR1 functions as a negative regulator of YTHDF2‐mediated m^6^A recognition in response to HHT treatment. To this end, we synthesized biotinylated RNA probes and m^6^A probe to perform RNA pull‐down analysis. Our data demonstrated a significant enrichment of YTHDF2 in cells, which was notably reduced following HHT treatment (Figure 6H), whereas the binding of other m^6^A reader proteins, including IGF2BP1, YTHDF1, and YTHDF3, remained largely unaffected (Figure S11K). In addition, EWSR1 overexpression resulted in a reduced enrichment of YTHDF2 in the RNA pull‐down assay (Figure 6I).

To investigate the effect of HHT on global m^6^A RNA levels, we performed m^6^A dot blot assays in NB4 cells. As shown in Figure S12A, HHT treatment led to a concentration‐dependent increase in m^6^A level. The methylene blue (MB) staining confirmed equal RNA loading. Time‐course analysis further demonstrated that m^6^A accumulation was gradually enhanced following HHT treatment (Figure S12B). Quantification of m^6^A/A ratio through LC‐MS/MS confirmed a significant increase in m^6^A levels in a concentration‐dependent manner (Figure S12C). YTHDF2, a key m^6^A reader, normally binds to m^6^A‐modified mRNAs and promotes their degradation. Disruption of the EWSR1‐YTHDF2 interaction by HHT impairs this function, preventing YTHDF2 from recognizing and degrading m^6^A‐modified RNAs, which leads to the accumulation of these transcripts. Thus, the observed global increase in m^6^A levels is likely a secondary effect of YTHDF2 dysfunction (due to retained m^6^A‐modified transcripts). Taken together, our findings strengthen the evidence that EWSR1 is a critical target of HHT in modulating the m^6^A recognition function of YTHDF2.

### HHT stabilizes m^6^A‐modified transcripts via EWSR1‐YTHDF2 axis

**Figure 7:**
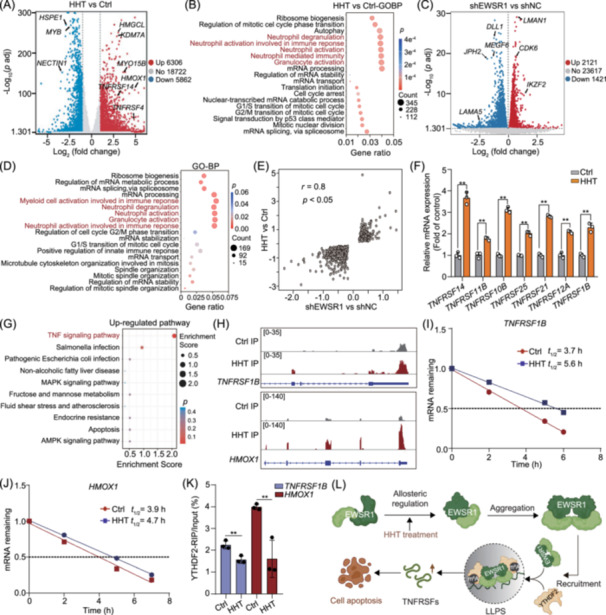
HHT modulates m^6^A‐modified RNA stability through the EWSR1‐YTHDF2 axis. (A) Volcano plot of up‐ or downregulated genes in NB4 cells treated with HHT. (B) GO enrichment analysis of differentially expressed genes in HHT‐treated NB4 cells. (C) Volcano plot showing the differentially expressed genes in shEWSR1 versus shNC NB4 cells. (D) Bubble chart showing the Gene Ontology (GO) enrichment analysis of differentially expressed genes in shEWSR1 versus shNC NB4 cells. (E) Scatter‐plots illustrating the correlation of gene expression alterations following treatment with EWSR1 siRNA or HHT at 40 nM. (F) qRT‐PCR analysis of differentially expressed mRNAs in NB4 cells with or without HHT treatment (1 technical replicate of *n* = 3 biological replicates per group). (G) Kyoto Encyclopedia of Genes and Genomes (KEGG) pathway enrichment analysis of upregulated genes with m^6^A modifications. (H) Alterations in the m^6^A modification of the *TNFRSF1B* and *HMOX1* genes following HHT treatment. (I, J) Decay curves of *TNFRSF1B* or *HMOX1* mRNAs in NB4 cells treated with HHT. (K) Enrichment of *TNFRSF1B* and *HMOX1* in YTHDF2 immunoprecipitants from NB4 cells treated with HHT. Bars represent mean ± SD; **p < 0.01. (L) Schematic representation of the EWSR1 phase transition mechanism regulated by HHT.

The involvement of EWSR1 in the modulation of RNA metabolism has been extensively documented [31, 32, 33]. Therefore, RNA‐sequencing (RNA‐seq) was performed on NB4 cells. The volcano plot demonstrated a significant upregulation of 6036 genes and downregulation of 5862 genes (Figure 7A). To explore the variations in signaling pathways, we performed Kyoto Encyclopedia of Genes and Genomes (KEGG) and Gene Ontology (GO) pathway enrichment analyses on the genes that showed differential expression. KEGG enrichment analysis showed the differentially expressed genes were mainly enriched in the mitophagy, cell cycle, and RNA degradation (Figure S13A). Meanwhile, GO BP category analysis indicated that the biological processes are associated with ribosome biogenesis, autophagy, neutrophil degranulation, neutrophil activation, mRNA processing, and mRNA splicing (Figure 7B). In addition, we performed a transcriptomic analysis to determine whether EWSR1 knockdown highly correlated with the transcriptional features observed with HHT treatment. Here, we generated EWSR1 knockdown NB4 cells for RNA‐seq analysis. As a result, we identified a total of 2121 upregulated genes and 1421 downregulated genes (Figure 7C). Furthermore, we observed significant enrichment in protein processing in the endoplasmic reticulum, as well as RNA degradation and RNA degradation pathways (Figure S13B). GO enrichment analysis revealed a substantial enrichment of biological processes associated with ribosome biogenesis, neutrophil degranulation, neutrophil activation, mRNA processing, and mRNA splicing in EWSR1 knockdown cells (Figure 7D). In particular, the expression changes of about 2872 genes in HHT‐treated cells showed a significant overlap and strong correlation with those observed in EWSR1 knockdown cells, providing additional evidence that EWSR1 is a functional target of HHT (Figure 7E).

The tumor necrosis factor (TNF) and TNF receptor (TNFR) superfamilies (TNFSF/TNFRSF) play a vital role in immune response and programmed cell death. In this study, we observed a significant upregulation of *TNFRSF14*, *TNFRSF11B*, *TNFRSF10B*, *TNFRSF25*, *TNFRSF21*, *TNFRSF12A*, and *TNFRSF1B* in NB4 cells treated with HHT (Figure 7F, Table S1). To further explore the downstream target genes of YTHDF2, we conducted methylated RNA immunoprecipitation sequencing (MeRIP‐seq) on NB4 cells (Figure S13C). KEGG pathway enrichment analysis revealed that upregulated m^6^A‐modified genes were primarily involved in the TNF signaling pathway (Figure 7G), while downregulated m^6^A‐modified genes were significantly associated with autophagy and the AMPK signaling pathway (Figure S13D). Our findings revealed that HHT significantly increased the m^6^A modification levels of *TNFRSF1B* and *HMOX1* mRNAs in leukemia cells (Figure 7H, Figure S13E). Based on this, we hypothesized that HHT might influence the stability of *TNFRSF1B* and *HMOX1* mRNAs. qRT‐PCR analysis further demonstrated that HHT inhibited the degradation of *TNFRSF1B* and *HMOX1* mRNAs while reducing their association with YTHDF2 (Figure 7I–K). Taken together, our findings suggest that HHT enhances the interaction between EWSR1 and YTHDF2, thus inhibiting the recognition of the m^6^A‐modified target genes *TNFRSF1B* and *HMOX1*, ultimately leading to apoptosis in leukemia cells (Figure 7L).

### EWSR1 serves as a therapeutic target for AML in vivo

**Figure 8:**
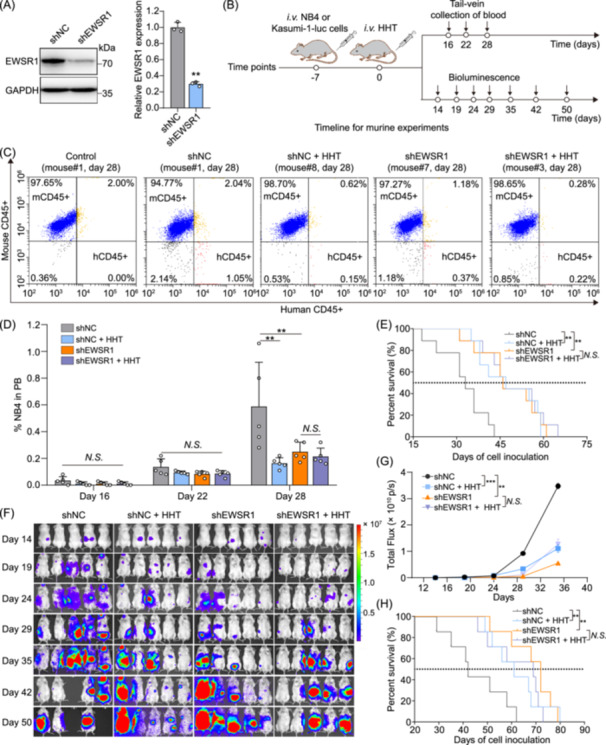
Targeting EWSR1 with HHT suppresses AML progression in vivo. (A) Western blot and relative quantification of EWSR1 expression following infection with shNC or shEWSR1 lentiviral particles. **p < 0.01, *n* = 3. (B) Schematic of the in vivo experimental workflow. Mice were intravenously injected with NB4 or Kasumi‐1‐luc cells on Day −7, followed by treatment with HHT starting on Day 0. (C) Peripheral blood (PB) samples were collected at the indicated time points and analyzed for NB4 cells (human CD45+) by flow cytometry. Representative results from Day 28 are shown. (D) Quantification of NB4 cells in PB on Days 16, 22, and 28 post‐transplantation in mice from the indicated groups. (E) Kaplan–Meier survival curves of mice transplanted with NB4 cells, analyzed by Mantel–Cox (log‐rank) test. (F) Representative bioluminescent images showing tumor burden in NOG mice transplanted with Kasumi‐1‐luc cells from the indicated groups (shNC, shNC + HHT, shEWSR1, shEWSR1 + HHT) at Days 14, 19, 24, 29, 35, 42, and 50 postinoculation. The radiance is shown on a color scale (Min = 7.54e^4^, Max = 1.99e^7^ p/sec/cm²/sr). (G) The quantification of total flux (p/s) in mice transplanted with Kasumi‐1‐luc cells, showing progressive tumor growth with significant differences between treated and control groups. Data were analyzed by two‐way repeated measures ANOVA with Dunnett's multiple comparisons test. (H) Kaplan–Meier survival curves of the mice transplanted with Kasumi‐1‐luc cells, showing significant differences in survival between groups. Bars represent mean ± SD; **p < 0.01, ***p < 0.001; *N.S*., not significant. “shNC” represents control group, “shEWSR1” indicates EWSR1 knockdown, and “HHT” denotes HHT treatment.

To validate the role of HHT in mediating anti‐AML effects via EWSR1 in vivo, we utilized lentiviral transduction to establish stable EWSR1‐knockdown (shEWSR1) and control (shNC) NB4 and Kasumi‐1 cell lines, confirmed by Western blot analysis showing ~70% EWSR1 reduction (Figure 8A). These two cell lines were used to construct distinct AML models in immunodeficient mice. NB4 cells were transplanted into NOD/SCID mice to establish a model for quantifying human leukemia cell infiltration via flow cytometric analysis. Luciferase‐labeled Kasumi‐1 cells were transplanted into NOG mice to generate a model enabling longitudinal tracking of leukemia progression via bioluminescence imaging (BLI). HHT (0.5 mg/kg) was administered via tail vein injection for four consecutive weeks, with longitudinal monitoring of leukemia progression, and the survival rate was continuously monitored (Figure 8B). Blood samples were collected from the mice through the tail vein to perform CD45 immunofluorescence staining. Flow cytometric analysis revealed the presence of NB4 clonal expansion (human CD45+) in the peripheral blood (PB) on Day 28, comprising over 0.5% of PB cells. The average proportion of NB4 cells in PB was 0.58% in the control group and 0.16% in wild‐type EWSR1 mice administered with HHT on day 28 (Figure 8C,D). Meanwhile, in the EWSR1 knockdown mice, on Day 28, the average proportions of NB4 cells in PB were 0.25% for the control mice and 0.21% for the mice treated with HHT. Moreover, survival curve analysis of the mice demonstrated that in the wild‐type EWSR1 mouse model, treatment with HHT displayed a notable therapeutic effect against AML, leading to a significant extension in the lifespan of the mice. On the contrary, in the EWSR1 knockdown mouse model, extension in the lifespan of the mice was observed, and HHT could not further increase the lifespan of the mice (Figure 8E). The extended lifespan of mice administered HHT was negatively correlated with the clonal expansion of NB4 cells in PB. Consistent findings were obtained in the Kasumi‐1 model. BLI revealed that tumor burden increased rapidly in the shNC group, while both HHT treatment and EWSR1 knockdown significantly suppressed leukemia progression. However, combined HHT treatment and EWSR1 depletion did not further reduce tumor growth compared to either intervention alone, suggesting that EWSR1 is required for HHT efficacy (Figure 8F). Quantification of bioluminescence signals confirmed these findings (Figure 8G). Consistently, survival analysis demonstrated that both HHT treatment and EWSR1 knockdown significantly prolonged survival compared to control group, but their combination did not provide additional survival benefits (Figure 8H). Taken together, these results strongly indicate that EWSR1 acts as a direct target of HHT, playing a crucial role in its anti‐AML effects in vivo.

## DISCUSSION

Currently, the clinical treatment for AML, especially APL, has been a combination of arsenic trioxide and all‐trans retinoic acid [34, 35]. However, there are still patients who experience a recurrence of the disease after this treatment. Therefore, in clinical practice, while applying the combination therapy of arsenic trioxide and all‐trans retinoic acid, chemotherapy drugs are often used for synergistic treatment as well. HHT is a widely employed therapeutic agent for the treatment of leukemia, demonstrating efficacy against various leukemia subtypes [36]. In particular, in treating APL, the combination of HHT with arsenic‐based therapies is typically employed to significantly reduce the disease recurrence. Therefore, by identifying the direct targets of HHT and subsequently determining the expressions of target genes in AML patients, we can ascertain which populations exhibit heightened sensitivity to HHT. In this study, we employed chemical biology techniques to identify EWSR1 as the direct molecular target of HHT in AML cells. This finding elucidates the close association between elevated EWSR1 expression and disease progression in AML. Meanwhile, the elevated expression levels of EWSR1 are more strongly associated with responsiveness to HHT. Therefore, our results indicate that genetic typing of patients during the clinical treatment of AML can potentially enhance the clinical efficacy of HHT.

A major strength of our target‐identification lies in the integration of two complementary, orthogonal approaches to validate HHT's protein targets: (1) a cell‐free, high‐throughput HuProt™ human proteome microarray, which profiles binding to purified recombinant proteins, and (2) a cellular SILAC‐labeled pull‐down assay, which captures protein interactions within the native physiological environment of leukemia cells. This dual strategy was designed to minimize false positives from simplified biochemical systems while ensuring biological relevance in live cells. The HuProt™ microarray initially identified 42 candidate proteins with strong HHT binding (SNR > 3), from which we prioritized the top 10 hits with the highest SNR values, including EWSR1, PSMA3, LRRC1, HNRNPD, and FAM98A. Subsequent SILAC‐based pull‐down assays in NB4 cells confirmed EWSR1 as the most significantly enriched target, with a light/heavy ratio ~2.5‐fold higher than that of the next candidate. The consistent ranking of EWSR1 among the top hits in both in vitro and cellular assays provides compelling evidence that it is the primary target of HHT. By contrast, proteins such as PSMA3 and LRRC1, which exhibited strong binding in the microarray, showed weaker enrichment under native conditions, underscoring the limitations of purely in vitro approaches and the importance of cellular validation. Together, these orthogonal findings support the prioritization of EWSR1 as the key functional mediator of HHT activity in leukemia cells.

Previous studies have revealed that EWSR1 exhibits the characteristic of phase separation [37]. However, the exact link between EWSR1 phase separation and RNA m^6^A modification, and their respective roles in driving the pathogenesis of AML, remain incompletely comprehended. In this study, we discovered that EWSR1 phase separation recruits YTHDF2, thereby regulating the stability of a series of RNAs in AML cells. Our study emphasizes a promising chemical strategy using HHT as a molecular probe to selectively induce EWSR1 phase separation. The stability of RNAs is effectively regulated through a m^6^A‐YTHDF2‐dependent mechanism, underscoring its potential for therapeutic applications. Therefore, these findings provide valuable insights for the development of targeted therapies for AML and enhance our understanding of its molecular mechanisms.

The clinical evidence suggests a strong association between elevated levels of *EWSR1* gene expression and decreased patient survival rates as well as unfavorable prognoses [38, 39], highlighting *EWSR1* as a potential target for cancer treatment. Here, we illustrated that the capacity of HHT to promote the phase separation of EWSR1 in cells resulted in the recruitment of YTHDF2 from protein droplets, ultimately hindering the progression of AML. Therefore, this study presents a novel therapeutic strategy for AML by targeting the EWSR1‐YTHDF2 axis. Given that HHT can enhance the effects of arsenic‐based combination therapy in clinical settings, we speculate that the EWSR1‐YTHDF2 axis may play a synergistic role with arsenic trioxide and all‐trans retinoic acid. In addition, to our knowledge, there are currently no reports on the involvement of arsenic trioxide or all‐trans retinoic acid in the EWSR1‐mediated signaling mechanisms. Therefore, EWSR1 may provide molecular biological evidence to reveal the synergistic effects of HHT.

Additionally, our study unveils a mechanism by which HHT does not directly increase the expression of EWSR1, but rather facilitates the process of phase separation. The investigation has elucidated that the initiation of EWSR1 phase separation is primarily mediated by its self‐polymerization process. Moreover, this process is facilitated by multiple interactions with essential m^6^A‐associated proteins, particularly YTHDF2. Here, we suggest that the aggregation of EWSR1 is probably augmented by hydrogen bonding interactions among specific amino acids present within the RNA recognition motif of EWSR1. Hence, these interactions are essential for facilitating the prompt assembly of polymer, which in turn triggers the phase separation of EWSR1. The precise binding site of HHT with EWSR1 can be found within the C‐terminal RNA‐binding domain, which is known to be responsible for the phase separation of EWSR1. Consequently, HHT possesses the potential to promote the binding of two EWSR1 proteins by establishing specific hydrogen bonds with particular amino acids within this domain. The formation of specific hydrogen bonds is crucial for creating of a ternary complex that involves EWSR1 proteins and HHT. In this study, we attempted to determine the structure of the HHT‐EWSR1 complex, but were hindered by the intrinsic biophysical properties of EWSR1. We found that the extensive IDRs of EWSR1, particularly within the N‐terminal low‐complexity domain, generate conformational heterogeneity, thereby impeding stable crystal lattice formation. This plasticity, combined with LLPS, leads to the aggregation of dynamic condensates rather than enabling crystallization. Thus, these challenges reflect broader limitations in the structural characterization of IDR/LLPS‐containing proteins.

## CONCLUSION

In summary, our research identifies EWSR1 as a crucial target for HHT, thereby playing a role in alleviating the process of AML. Moreover, HHT regulates the phase separation of EWSR1, thereby enhancing our understanding of the fundamental role of the EWSR1‐YTHDF2‐m^6^A axis in the development of AML. Our study establishes a mechanistic foundation for the clinical application of HHT and suggests strategies to enhance its therapeutic potential in genetically diverse AML patients.

## METHODS

### Human sample collection and cell lines

Bone marrow mononuclear cells were collected from 11 healthy donors (HC group) and 26 AML patients. The human leukemia NB4 cell line (from a female donor) was kindly donated by Prof. Guo‐Qiang Chen of the Shanghai Jiao Tong University School of Medicine. HL‐60 cells (female donor) and human embryonic kidney (HEK) 293T cells (female donor) were sourced from the Cell Resource Center of Peking Union Medical College. K562, THP‐1, Kasumi‐1, OCI‐AML2, and MOLM13 cells were obtained from the ATCC, while the luciferase‐expressing Kasumi‐1 cell line (Kasumi‐1‐luc) was purchased from FuHeng Biology. The cells underwent testing using polymerase chain reaction (PCR) to ensure the absence of mycoplasma contamination. NB4 and HL‐60 cell lines were cultured in RPMI 1640 medium (Gibco, Life Technologies) supplemented with 10% fetal bovine serum (FBS, Gibco, Thermo Fisher Scientific), and antibiotics (100 mg/mL penicillin and 100 mg/mL streptomycin). HEK293T cells were grown in high‐glucose Dulbecco's Modified Eagle Medium (DMEM, Macgene), supplemented with 10% FBS and 1% penicillin/streptomycin. All cell cultures were incubated at 37°C in a 5% CO_2_, humidified atmosphere.

### Compound preparation

Homoharringtonine (HHT), with a purity of >98% from Baoji Herbest Bio‐Tech (Baoji), was used in the study. It was dissolved in dimethyl sulfoxide (DMSO) to create a stock solution at 20 mM, which was then stored at −20°C. Before each in vitro experiment, the HHT concentrate solution was adjusted with the appropriate medium to obtain the desired concentration. The concentration of DMSO in the experimental medium was maintained below 0.1%, ensuring its minimal impact on the study.

### Synthesis of bio‐HHT probe

Compound HHT reacted with propargyl acid under esterification conditions using dicyclohexyl‐carbodiimide (DCC) and 4‐pyrrolidinopyridine (4‐ppy) at 35°C, leading to the formation of HHT‐HEX. To prepare the reaction mixture, CuSO_4_ (8 mM), azide‐PEG_3_‐biotin (100 mM), and HHT‐HEX (50 mM) were dissolved in 1.5 mL of click buffer containing 800 μM tris((1‐benzyl‐4‐triazolyl)methyl)amine (TBTA), 8 mM tris(2‐carboxyethyl)phosphine (TCEP), and 40% *t*‐butyl alcohol (*t*‐BuOH). Nitrogen gas was introduced into the mixture, and the final solution was incubated at a temperature of 4°C overnight. For the separation of the reaction product, thin‐layer chromatography was employed. A two‐phase solvent system consisting of dichloromethane (DCM) and methanol (MeOH) was utilized, resulting in a effective separation. To determine the chemical structure of bio‐HHT, nuclear magnetic resonance (NMR) techniques and high‐resolution mass spectrometry were employed.

### Synthesis of HHT‐PAL probe

The following reagents were added to a 2 ml solution of dichloromethane (DCM): 50 mg of HHT, 22 mg of 3‐(3‐(but‐3‐yn‐1‐yl)−3H‐diazirin‐3‐yl)propanoic acid (BDPA), 37 mg of 1,3‐dicyclohexylcarbodiimide (DCC), and 27 mg of 4‐pyrrolidinopyridine (4‐ppy). The final solution was heated at 35°C for 12 h. Silica gel column chromatography was used to separate the reaction product. A DCM and MeOH solvent system was used, resulting in successful separation. High‐resolution electrospray ionization mass spectrometry (HRESIMS), ^1^H NMR, and ^13^C NMR techniques were utilized for the determination of the chemical structure of HHT‐PAL.

### Immunoblotting

The protein lysates were mixed with protein loading buffer, containing 125 mM Tris‐HCl, pH 6.8, 20% SDS, 10% glycerol, 0.5% beta‐mercaptoethanol, and 0.5% bromophenol blue, and heated at 98°C for 10 min. The protein samples were resolved on SDS‐PAGE gels and subsequently transferred to polyvinylidene difluoride membranes (0.45 μm). To prevent nonspecific binding, the membranes were blocked using 5% skimmed milk powder. Next, the membranes were incubated with primary antibodies targeting specific proteins at the following dilutions: anti‐GAPDH (1:5000, Proteintech), anti‐EWSR1 (1:1000, Proteintech), anti‐HA (1:1000, Cell Signaling Technology), anti‐His (1:1000, Cell Signaling Technology), anti‐YTHDF2 (1:1000, Proteintech), and anti‐Myc (1:1000, Cell Signaling Technology). After incubation with the primary antibodies, the membranes were exposed to secondary antibodies at a 1:5000 dilution. Finally, the protein bands on the membranes were visualized using Super Signal™ West Femto Maximum Sensitivity Substrate (Thermo Fisher Scientific).

### Human proteome microarray analysis

HuProt™ Human Proteome Microarray v3.1 was obtained from CDI Laboratories, Inc (Mayaguez, PR). The proteome microarrays were incubated with blocking solution (3% BSA in PBS) on a shaker at 60 rpm for 1 h. After blocking, the microarrays were promptly incubated with a preprepared sample incubation liquid containing a concentration of 10 μM bio‐HHT for 1 h. Subsequently, the microarrays were rinsed three times with PBST. Then, a 1:1000 dilution of Cy3‐streptavidin was prepared and added to the proteome microarray. After washing the microarrays with PBST buffer, they were rinsed with ddH_2_O and spun dry. The results were visualized by a GenePix 4200A microarray scanner (Axon Instruments). The signal‐to‐noise ratio (SNR) for microarrays was calculated by dividing the median foreground intensity by the median background intensity. The mean SNR was utilized as a representation of protein signal. To screen potential candidates, a cutoff value of ≥2.5 was set.

### Microscale thermophoresis (MST) analysis

The interaction between HHT and EWSR1 was assessed using the MST assay. HEK293T cells were transiently transfected with pEGFP‐N1‐EWSR1 to overexpress EWSR1‐GFP protein. The cells were lysed using NP40 buffer. Different concentrations of HHT, ranging from 500 μM to 15 nM, were serially diluted. The diluted HHT solutions were combined with lysates containing EWSR1‐GFP or GFP, then transferred into Monolith standard‐treated capillaries. Subsequently, the samples were monitored using a Monolith NT.115 instrument (Nano Temper Technologies) with MST power set at 40% and LED/excitation power at 20%. The *K*_D_ values were determined by employing the MO. Affinity Analysis software (Nano Temper Technologies).

### Cellular thermal shift assay (CETSA)

The cell lysate CETSA was conducted to investigate the direct interaction of HHT with EWSR1. HHT and EWSR1. NB4 cells were initially collected and lysed with NP40 buffer for 30 min. The lysates were then centrifuged at 12,000 × *g* for 10 min at 4°C, and subsequently divided into two separate aliquots. One aliquot was incubated with HHT at a concentration of 10 μM, while the other aliquot was treated with DMSO and incubated for 2 h. After the incubation period, the lysates were placed in reaction tubes and subjected to heat shock for 3 min using the T100 Thermal Cycler. The temperature range for heat shock was set from 37°C to 78°C. Finally, all the samples were boiled with loading buffer for immunoblotting analysis.

### Drug affinity responsive target stability (DARTS) assays

Cells were collected and lysed with NP‐40 buffer containing 1 mM PMSF for 30 min. The lysates were subsequently mixed with TNC solution (500 mM Tris‐HCl, pH 8.0, 100 mM CaCl_2_, 500 mM NaCl) and incubated with HHT at different concentrations (0, 0.1, 0.2, 0.4 μM) for 1 h. Following the incubation, 2 μg/mL pronase (Roche Diagnostics GmbH) was added to the lysates and further incubated for 20 min at room temperature. The reaction was stopped by adding protein loading buffer, and the samples were incubated for 10 min at 98°C. Protein samples were subsequently subjected to analysis through western blot using an anti‐EWSR1 antibody.

### Plasmid construction

A human EWSR1 full‐length sequence (amino acids 1‐656) was commercially synthesized and constructed into pCMV‐HA vector by Generay Biotech. The truncations of EWSR1, including EWSR1‐N (amino acids 1‐300), EWSR1‐C (amino acids 301‐656) plasmids were constructed into pCMV‐HA vector by homologous recombination method. The EWSR1, EWSR1‐N, and EWSR1‐C were further constructed into pEGFP‐N1 vector to generate the EWSR1‐EGFP, EWSR1‐N‐GFP, and EWSR1‐C‐GFP expressing plasmids. Sequences for mCherry and Cry2WT were inserted into the pcDNA3.1 vector to generate the pcDNA3.1‐mCherry‐Cry2WT plasmid. For constructing optoDroplet system, DNA fragments encoding the EWSR1 and truncations were inserted into the linearized pcDNA3.1‐mCherry‐Cry2WT vector by pEASY‐Basic Seamless Cloning and Assembly Kit (Trans). Moreover, EWSR1‐C (amino acids 301‐656) was cloned into a pGEX‐4T‐1 vector with a GST tag sequence at the N‐terminal region. The resulting constructs were confirmed by Sanger sequencing (RuiBiotech).

### Pull‐down assay

Cells were transfected with various HA‐tagged EWSR1 plasmid constructs, including full‐length (HA‐EWSR1), N‐terminal (HA‐EWSR1‐N), C‐terminal (HA‐EWSR1‐C), and point mutants (HA‐EWSR1‐M397A, HA‐EWSR1‐T393A, HA‐EWSR1‐P396A, HA‐EWSR1‐I400A, and HA‐EWSR1‐Mut4). After treatment for 48 h, cells were harvested and lysed with NP40 lysis buffer along with the addition of PMSF, a protease inhibitor, for 30 min. The cell lysates were centrifuged at 12,000 rpm for 15 min at 4°C. Bio‐HHT was incubated with streptavidin magnetic beads for 2 h. Subsequently, the cell lysates were incubated with the HHT‐coupled beads, along with control beads, overnight at 4°C. After incubation, the beads were rinsed with PBS to eliminate nonbinding components. Binding proteins were eluted with loading buffer and analyzed through western blot.

### BiFC analysis

The EWSR1 was fused with the N‐terminal fragment of mVenus (VN1‐173) and C‐terminal fragment of mVenus (VC155‐238). Subsequently, these fused constructs were cloned into the pcDNA3.1 expression vector. For BiFC assays, HEK293T cells at 60%–80% confluency were transfected with plasmids via linear polyethylenimine (PEI). After 24 h, the cells were fixed with 4% formaldehyde, incubated with 1 μg/mL of Hoechst 33342 for 10 min, and then rinsed with PBS. Subsequently, the cells were visualized by a Zeiss LSM880 laser scanning confocal microscope.

### Surface plasmon resonance (SPR) analysis

We employed SPR with the Biacore T200 system (GE Healthcare) to investigate the biomolecular interaction between HHT and EWSR1. Sulpho‐NHS/EDC chemistry was employed to initiate the activation of the CM5 sensor chip. Next, the recombinant human EWSR1 protein (Sino Biological) was diluted in 10 mM sodium acetate (pH 4.5) and immobilized onto the chip. Ethanolamine was used to block the CM5 chip. HHT was then diluted in PBS‐P buffer (with 5% DMSO, v/v) and injected at a flow rate of 20 μL/min in the running buffer. Data analysis was performed with Biacore Evaluation Software (T200 Version 2.0). The binding between HHT and EWSR1 was analyzed using the steady‐state affinity model.

### Gene knockdown

The siRNA sequences targeting *EWSR1* (siEWSR1, 5′‐CGCCUAUGCAACUUCUUAUTT ‐3′) and the control siRNA were obtained from Gene Pharma (Suzhou, Jiangsu, China). The lenti‐shEWSR1 vector and their corresponding control vector were purchased from Hanbio. For stable knockdown, NB4 cells were transduced with lentiviral particles at a multiplicity of infection of 40. After 24 h, the culture medium was replaced, and cells were selected using 5 μg/mL puromycin for 14 days.

### SILAC media preparation and cell culture conditions

The cell culture was conducted using the standard stable isotope labeling with amino acids in cell culture (SILAC) method, as previously described [40, 41]. The base media, DMEM (Macgene), was divided into two portions: one supplemented with l‐arginine (Arg°) and l‐lysine (Lys°) for the light condition, and the other with ^13^C_6_^15^N_4_‐l‐arginine (Arg^10^) and ^13^C_6_^15^N_2_‐l‐lysine (Lys^8^) for the heavy condition, generating two distinct SILAC isotope‐labeled mediums. Cells were cultured in the respective labeling medium, supplemented with antibiotics and 10% dialyzed FBS, and maintained for at least six cell divisions in a humidified atmosphere at 37°C with 5% CO_2_.

### Co‐immunoprecipitation assay

HEK293T cells cultured in SILAC medium were transfected with HA‐EWSR1 plasmid and treated with HHT (40 nM). Cells were lysed in NP40 buffer supplemented with 1 mM PSMF for 30 min, followed by centrifugation for 10 min. The supernatant was incubated with HA‐tag magnetic beads (Cell Signaling Technology) at 4°C for 4 h. Beads were washed with PBS, and bound proteins were analyzed by mass spectrometry and validated by western blot analysis with the indicated antibodies.

### Expression and purification of recombinant proteins

The DNA sequence encoding EWSR1‐C was cloned into the pET‐32a (+) vector at the EcoR I and Xho I restriction sites and transformed into *Escherichia coli* BL21 (DE3). The strain was cultured in Lysogeny broth (LB) medium containing 50 µg/mL of ampicillin at 37°C. When the optical density reached 0.8, protein expression was induced by adding 500 μM isopropyl‐*β*‐D‐1‐thiogalactopyranoside (IPTG) at 16°C for 12 h. Cells were harvested by centrifugation at 9000 rpm for 10 min, lysed in buffer A (20 mM Hepes, 250 mM NaCl, and 10 mM imidazole, pH 7.5) by sonication, and clarified by centrifugation at 8000 rpm for 40 min at 4°C. The supernatant was applied to Ni‐NTA beads (Qiagen), washed with buffer B (20 mM Hepes, 250 mM NaCl, and 50 mM imidazole, pH 7.5), and eluted with buffer C (20 mM Hepes, 250 mM NaCl, and 250 mM imidazole, pH 7.5). The purified proteins were concentrated by centrifugal filtration (Amicon Ultra‐15 mL, Millipore), analyzed for purity using 12% (w/v) SDS‐PAGE, and preserved in PBS at −80°C.

### HDX‐MS analysis

The EWSR1‐C protein was pre‐equilibrated with HHT solution for 30 min at room temperature (RT) and then diluted 20‐fold in D_2_O buffer for HDX labeling. Reactions were quenched at the indicated times with a quenching buffer containing 100 mM phosphate, 4 M guanidine hydrochloride, 0.5 M TCEP, pH 2.0. Samples were subjected to online enzymatic digestion using a Waters ENZYMATE BEH pepsin column (2.1 mm × 30 mm, 5 μm). Subsequently, the peptides were then trapped and desalted for 3 min on a VanGuard Pre‐Column Trapping Cartridge (ACQUITY UPLC BEH C18, 1.7 μm) with 2% acetonitrile at a flow rate of 100 μL/min for elution. Then, the samples were separated on an ACQUITY UPLC BEH C18 column (1.7 μm, 1.0 mm × 100 mm). Relative deuterium incorporation was determined by comparing the mass difference between labeled samples and unlabeled controls.

### m^6^A dot blot analysis

The m^6^A dot blot assay was performed as previously described with modifications [42]. Poly(A)+ mRNA was denatured at 65°C for 5 min using RNA incubation buffer (1.33 × MOPS, 65.7% formamide, and 7.77% formaldehyde), followed by the addition of an equal volume of chilled SSC buffer. Samples were spotted onto Amersham Hybond‐N+ membranes using a Bio‐Dot apparatus and UV‐crosslinked. Membranes were blocked with 5% nonfat dry milk and incubated overnight at 4°C with anti‐N^6^‐Methyladenosine (m^6^A) antibody (1:1000, Cell Signaling Technology). Signals were detected using enhanced chemiluminescence (ECL) substrate and imaged with a Tanon 5200 Imaging Analysis System (Tanon). Membranes were counterstained with methyl blue and imaged for RNA loading control.

### Quantitation of m^6^A levels by LC‐MS/MS

The ratio of m^6^A/A was measured by HPLC‐MS/MS as previously described [43]. Total RNA was extracted using the MolPure® Cell/Tissue Total RNA Kit (Yeasen), and mRNA was further purified with Hieff NGS® mRNA Isolation Master Kit mRNA (Yeasen). Approximately 60 ng of RNA was digested with 1 unit of Nuclease P1 at 42°C for 2 h, followed by incubation with 100 mM ammonium bicarbonate and 1 unit of alkaline phosphatase (Sigma, P5931) at 37°C for 2 h. After fivefold dilution, 20 μL of each sample was analyzed by HPLC‐MS/Mm6AS. Quantitation was performed using nucleoside‐to‐base ion transitions (268‐to‐136 for adenosine (A); 282‐to‐150 for N6‐methyladenosine (m^6^A)). Standard curves from pure nucleosides were used to calculate A and m^6^A concentrations.

### RNA stability assays

NB4 cells were treated with 5 μg/mL actinomycin D and 40 nM HHT for 2, 5, or 6 h, after which total RNA was isolated using MolPure® Cell/Tissue Total RNA Kit (Yeasen). Relative mRNA levels were quantified by RT‐qPCR on a Roche LightCycler® 96 system. The mRNA turnover rate and half‐life were determined as previously described [44], with actinomycin D blocking transcription so that a decrease in mRNA levels reflects degradation. The decay rate constant *K*_decay_ was determined by the following equation:

$$\mathrm{In}\left(C/C_{0}\right)=-K_{\mathrm{decay}}t.$$

The equation relates the natural logarithm of the ratio of mRNA concentration at time *t* (*C*) to the initial mRNA concentration (*C*_0_) at time 0 h to the degradation rate constant (*K*_decay_) and time (*t*). By plotting In (*C/C*_0_) versus time (*t*) and fitting the data with a linear regression, we can determine the value of *K*_decay_. The half‐time (*t*_1/2_) represents the time at which the mRNA concentration (*C*) decreases to half of its initial value (*C*_0_), i.e., *C*/*C*_0_ = ½, and can be calculated using the following equation:

$$\mathrm{In}\left(1/2\right)=-K_{\mathrm{decay}}t_{1/2}.$$

From where:

$$t_{1/2}=\mathrm{In}2/K_{\mathrm{decay}}.$$

### Methylated RNA immunoprecipitation sequencing (MeRIP‐seq)

The m^6^A MeRIP‐Seq was conducted in collaboration with CloudSeq (Shanghai, China). Total RNA was extracted, and ribosomal RNA was removed to enrich non‐ribosomal RNA species. The RNA was then fragmented into approximately 200 nucleotide fragments using RNA Fragmentation Reagents. To capture methylated RNA, protein A/G magnetic beads were coated with an m^6^A‐specific antibody (GenSeq m^6^A MeRIP Kit, GenSeq), and incubated with the fragmented RNA at 4°C for 4 h. The RNA/antibody complexes were washed to remove nonspecifically bound RNA, and the enriched RNA was eluted and purified. RNA libraries for both immunoprecipitated and input samples were generated using the GenSeq Low Input Whole RNA Library Prep Kit, according to the manufacturer's instructions. Library quality was assessed using an Agilent 2100 Bioanalyzer before sequencing.

### Bioinformatics analysis of AML in TCGA database

TCGA is a comprehensive data resource widely used for cancer bioinformatics analysis [45]. GEPIA2 (http://gepia2.cancer-pku.cn) is a web‐based tool specifically designed to analyze clinical data from TCGA to assess the correlation between gene expression levels and patient survival in various cancer types [46]. The expression profile of *EWSR1* was thoroughly examined in different types of malignancies, in comparison to normal tissues. This investigation was conducted using data obtained from the GEPIA database. Furthermore, a survival analysis was performed on genes displaying either high or low expression levels through the GEPIA2 website.

### Murine xenografting model of leukemia

Eight‐week‐old immunodeficient mice (NOD/SCID or NOG) were intravenously injected via the tail vein with 1 × 107 human leukemia cells (NB4 cells for NOD/SCID; Kasumi‐1‐luc cells for NOG) on day 1. Beginning on Day 7, mice received intravenous injection of HHT (0.5 mg/kg in 0.1 mL saline) through the tail vein once daily. For the NOD/SCID‐NB4 model, peripheral blood (PB) samples were obtained from the tail vein every 3–4 days, starting from day 16 until Day 28. To remove remaining red blood cells, erythrocyte lysis buffer (NaHCO_3_ 10 mM, NH4Cl 1.5 mM, and EDTA‐2Na 1 mM) was utilized. The isolated cells were then double‐stained with FITC‐conjugated anti‐human CD45 (BD Pharmigen) and PerCP‐Cy5.5‐conjugated anti‐mouse CD45 (BD Pharmigen) antibodies for subsequent flow cytometric analysis. In the NOG‐Kasumi‐1‐luc model, tumor progression was monitored by BLI (IVIS Spectrum, PerkinElmer) on days 14, 19, 24, 29, 35, 42, and 50 following intraperitoneal injection of d‐luciferin (150 mg/kg). Signal intensity was quantified as total photon flux (photons/sec/cm²/sr). Throughout the study, the lifespan of the mice was carefully observed, and the data were subjected to analysis using the Log‐rank (Mantel–Cox) test.

### Fluorescence recovery after photobleaching (FRAP)

HEK293T cells were transfected with pEGFP‐N1‐EWSR1 using PEI and cultured for 24 h. FRAP assay was performed on a Nikon A1R confocal microscopy equipped with a 60× oil objective and a 488 nm laser. Droplet regions of interest (ROIs) were photo‐bleached with 80% laser power for 4 s, and images were acquired every 1 s post‐bleaching. Fluorescence intensity recovery within the bleached ROIs was monitored over time, and the recovery rate and mobile fraction were quantified from intensity measurements. The average fluorescence recovery curve was generated data from three independent replicates.

### Single‐cell RNA‐seq (scRNA‐seq) data analysis

The scRNA‐seq count matrices from bone marrow samples of 20 healthy patients and 19 AML patients were downloaded from the GEO database (GSE120221 and GSE241989). To eliminate the influence of low‐quality cells, we applied the following filtering criteria: cells with fewer than 500 unique molecular identifiers, cells with mitochondrial gene expression exceeding 20%, and doublets detected using the scDblFinder (version 1.16.0) R package were excluded. The filtered data were processed with Seurat v4 (version 4.4.0) and batch effects were corrected with Harmony (version 1.2.0) before clustering. Normalization was performed using the NormalizeData function, and the top 4000 most variable genes were selected and scaled for principal component analysis. Using the first 25 principal components (PCs), dimensionality reduction was performed with unsupervised uniform manifold approximation and projection (UMAP), followed by cell clustering using a shared nearest neighbor modularity optimization algorithm at a resolution of 0.8. The resulting clusters were annotated based on the expression of bone marrow differentiation markers: HSC (CD34, AVP), Mast (TPSAB1, TPSB2), Monocyte (CD14/CD16), DC (CD1C), T (CD3D), Erythroid (HBD).

### Statistical analysis of proteomics data

Proteomics data were analyzed through a rigorous statistical pipeline to ensure reliable identification of differentially expressed proteins Raw intensities were log₂‐transformed, quantile‐normalized, and missing values imputed using a k‐nearest neighbors algorithm with a limit‐of‐detection assumption. Differential expression was assessed with a linear regression model and empirical Bayes moderation (limma, R), adjusting for batch effects and biological replicates. Proteins with FDR < 0.05 and |log₂FC| > 1.5 were considered significant. Functional enrichment was performed using GO and KEGG analyses with FDR‐adjusted hypergeometric tests. Correlation networks (|*r*| > 0.8, *p* < 0.01) and hierarchical clustering were applied to identify coregulated protein modules. All analyses were performed in R (v4.3.0) under stringent quality‐control standards.

### Statistical methods for correlation analysis

This study employed a comprehensive set of statistical approaches to assess the correlations between variables, with the Pearson correlation coefficient (*r*) serving as the core method for quantifying linear relationships. The Pearson correlation coefficient is designed to measure the strength and direction of the linear association between two continuous variables, calculated using the formula:

$$r=\frac{\sum_{i=1}^{n}\left(X_{i}-\bar{X}\right)\left(Y_{i}-\bar{Y}\right)}{\sqrt{\sum_{i=1}^{n}{\left(X_{i}-\bar{X}\right)}^{2}}\cdot \sqrt{\sum_{i=1}^{n}{\left(Y_{i}-\bar{Y}\right)}^{2}}},$$

where *X*_*i*_ and *Y*_*i*_ represent individual observations of the two variables, $\overset{®}{X}$ and $\overset{®}{Y}$ denote their respective sample means, and n is the total sample size. The coefficient ranges from [−1, 1], with interpretive thresholds defined as follows: ∣*r*∣ ≥ 0.8 indicates a strong linear correlation, while ∣*r*∣ ≤ 0.3 indicates a weak linear correlation. Values closer to 0 suggest little to no linear association.

### Quantification and statistical analysis

Statistical analyses were performed utilizing a combination of one‐way analysis of variance followed by Dunnett's multiple comparisons test, or Student's *t*‐test for pairwise comparisons. For single‐cell sequencing data and TCGA data analysis, the non‐parametric Wilcoxon rank‐sum test was employed. The number of replicates is specified in the figure legends and methods. Data are presented as mean ± standard deviation (SD) and differences were considered statistically significant at *p* < 0.05.

## AUTHOR CONTRIBUTIONS

**Ting‐Ting Liu**: Writing—original draft; funding acquisition; methodology; data curation. **Li‐Ting Chen**: Methodology; software. **Xu‐Ying Pei**: Resources; writing—review and editing. **Shao‐Nan Hu**: Investigation; writing—original draft. **Fang‐Fang Zhuo**: Project administration; data curation. **Ze‐Kun Chen**: Validation; methodology. **Yang Liu**: Investigation. **Jing‐Kang Wang**: Methodology; software. **Ji‐Chao Zhang**: Software; data curation. **Qi Cao**: Methodology; validation. **Ling Li**: Formal analysis; Software; data curation. **Jing Wang**: Software; methodology. **Tian‐Tian Wei**: Methodology; software; investigation. **Bo Han**: Software. **Peng‐Fei Tu**: Writing—original draft; writing—review and editing; validation. **Xiang‐Yu Zhao**: Writing—review and editing; data curation. **Ruidong Xue**: Formal analysis. **Ke‐Wu Zeng**: Writing—original draft; funding acquisition; investigation; writing—review and editing; supervision. All authors have read the final manuscript and approved it for publication.

## CONFLICT OF INTEREST STATEMENT

The authors declare no conflicts of interest.

## ETHICS STATEMENT

This study was conducted with the approval from the Ethics Review Committee of Peking University People's Hospital (Approval No. 2023PHB030‐001). Animal care and all experimental procedures received approval from the Institutional Animal Care and Use Committee of Peking University (License No. LA2022218).

## Supporting information


**Figure S1:** The synthesis route of bio‐HHT.
**Figure S2:** Structure identification of bio‐HHT.
**Figure S3:** Identification of HHT target proteins using pull‐down technology combined with stable isotope labeling by amino acids in cell culture (SILAC).
**Figure S4:** Single‐cell RNA sequencing reveals EWSR1 as a driver of AML progression.
**Figure S5:** EWSR1 expression and its association with HHT sensitivity in AML cells.
**Figure S6:** The interaction between HHT and EWSR1‐C recombinant protein was confirmed by pull‐down assay in vitro.
**Figure S7:** Structure identification of HHT‐PAL probe.
**Figure S8:** LC‐MS/MS analysis of covalently modified peptide of EWSR1‐C.
**Figure S9:** Pull‐down assays validate critical residues in the EWSR1 RRM domain for interaction with HHT.
**Figure S10:** HHT modulates liquid–liquid phase separation of EWSR1.
**Figure S11:** YTHDF2 is highly expressed in AML.
**Figure S12:** HHT treatment increases m^6^A methylation levels in NB4 cells.
**Figure S13:** HHT targets EWSR1 to regulate m^6^A‐modified pathways in leukemia cells.

**Table S1:** Primers for quantitative PCR.

## Data Availability

The data that support the findings of this study are openly available in GSA‐Human at https://ngdc.cncb.ac.cn/gsa-human/s/whx72O0s, reference number HRA013431. Public data used in this study are available in TCGA and GEO. Other datasets were obtained from published literature, as described in the Methods section. All the sequencing data have been deposited in GSA‐Human under submission number HRA013431 (https://ngdc.cncb.ac.cn/gsa-human/s/whx72O0s). All data and materials used in the analyses were included in the main text and supplementary. The data and scripts used are available at GitHub (https://github.com/ZKW246/ZKWiMeta). Supplementary materials (figures, tables, and graphical abstract) may be found in the online DOI or iMeta Science http://www.imeta.science/.
