# Supplementary material for: Homoharringtonine suppresses acute myeloid leukemia progression by orchestrating EWSR1 phase separation in an m6A‐YTHDF2‐dependent mechanism
Source: Imeta. 2025 Oct 30;4(6):e70089. doi: 10.1002/imt2.70089 (PMC12747546; doi:10.1002/imt2.70089)
Supplement: Supplementary file 1 — Figure S1: The synthesis route of bio‐HHT. Figure S2: Structure identification of bio‐HHT. Figure S3: Identification of HHT target proteins using pull‐down technology combined with stable isotope labeling by amino acids in cell culture (SILAC). Figure S4: Single‐cell RNA sequencing reveals EWSR1 as a driver of AML progression. Figure S5: EWSR1 expression and its association with HHT sensitivity in AML cells. Figure S6: The interaction between HHT and EWSR1‐C recombinant protein was confirmed by pull‐down assay in vitro. Figure S7: Structure identification of HHT‐PAL probe. Figure S8: LC‐MS/MS analysis of covalently modified peptide of EWSR1‐C. Figure S9: Pull‐down assays validate critical residues in the EWSR1 RRM domain for interaction with HHT. Figure S10: HHT modulates liquid–liquid phase separation of EWSR1. Figure S11: YTHDF2 is highly expressed in AML. Figure S12: HHT treatment increases m6A methylation levels in NB4 cells. Figure S13: HHT targets EWSR1 to regulate m6A‐modified pathways in leukemia cells. [file IMT2-4-e70089-s002.doc]

**Supporting information to**

**Homoharringtonine suppresses** **acute myeloid leukemia progression by orchestrating EWSR1 phase separation in an m^6^A-YTHDF2-dependent mechanism**

**Running title:** HHT suppresses AML via EWSR1-m^6^A-YTHDF2 axis

Ting-Ting Liu^1,2#^, Li-Ting Chen^3#^, Xu-Ying Pei^4#^, Shao-Nan Hu^5#^, Fang-Fang Zhuo^1^, Ze-Kun Chen^1,6^, Yang Liu^7^, Jing-Kang Wang^1^, Ji-Chao Zhang^1^, Qi Cao^3^, Ling Li^1^, Jing Wang^1^, Tian-Tian Wei^1^, Bo Han^8^, Peng-Fei Tu^1^*, Xiang-Yu Zhao^4^*, Ruidong Xue^1,3,9^*, Ke-Wu Zeng^1,6,8^*

^1^State Key Laboratory of Natural and Biomimetic Drugs, School of Pharmaceutical Sciences, Peking University, Beijing 100191, China.

^2^Institutes of Biomedical Sciences, School of Life Sciences, Inner Mongolia University, Hohhot 010070, China.

^3^Yunnan Baiyao International Medical Research Center, International Cancer Institute and State Key Laboratory of Molecular Oncology, MOE Frontiers Science Center for Cancer Integrative Omics, School of Basic Medical Sciences, Peking University, Beijing 100191, China.

^4^Peking University People’s Hospital, Peking University Institute of Hematology, National Clinical Research Center for Hematologic Disease, Beijing Key Laboratory of Cell and Gene Therapy for Hematologic Malignancies, Peking University, Beijing 100191, China.

^5^College of Pharmacy, Inner Mongolia Medical University, Hohhot 010110, China.

^6^Department of Integration of Chinese and Western Medicine, School of Basic Medical Sciences, Peking University, Beijing 100191, China.

^7^Center of Basic Medical Research, Institute of Medical Innovation and Research, Peking University Third Hospital, Beijing 100191, China.

^8^School of Pharmacy/Key Laboratory of Xinjiang Phytomedicine Resource and Utilization, Shihezi University, Shihezi 832003, China.

^9^Translational Cancer Research Center, Peking University First Hospital, Beijing 100191, China.

^#^These authors contributed equally: Ting-Ting Liu, Li-Ting Chen, Xu-Ying Pei, Shao-Nan Hu.

*Correspondence: pengfeitu@bjmu.edu.cn (Peng-Fei Tu), zhao_xy@bjmu.edu.cn (Xiang-Yu Zhao), rxue@hsc.pku.edu.cn (Ruidong Xue), ZKW@bjmu.edu.cn (Ke-Wu Zeng).

**Figure S1 The synthesis route of bio-HHT**. HHT undergoes an esterification reaction with propargyl acid in the presence of DCC and 4-ppy at 35 °C, resulting in the formation of HHT-HEX. Subsequently, the reaction mixture was prepared by dissolving CuSO_4_, azide-PEG_3_-biotin, and HHT-HEX in click buffer containing TBTA, TCEP, and *t*-BuOH, followed by nitrogen purging and incubation at 4 °C overnight. The product was separated using thin-layer chromatography (TLC) with a DCM/MeOH solvent system.


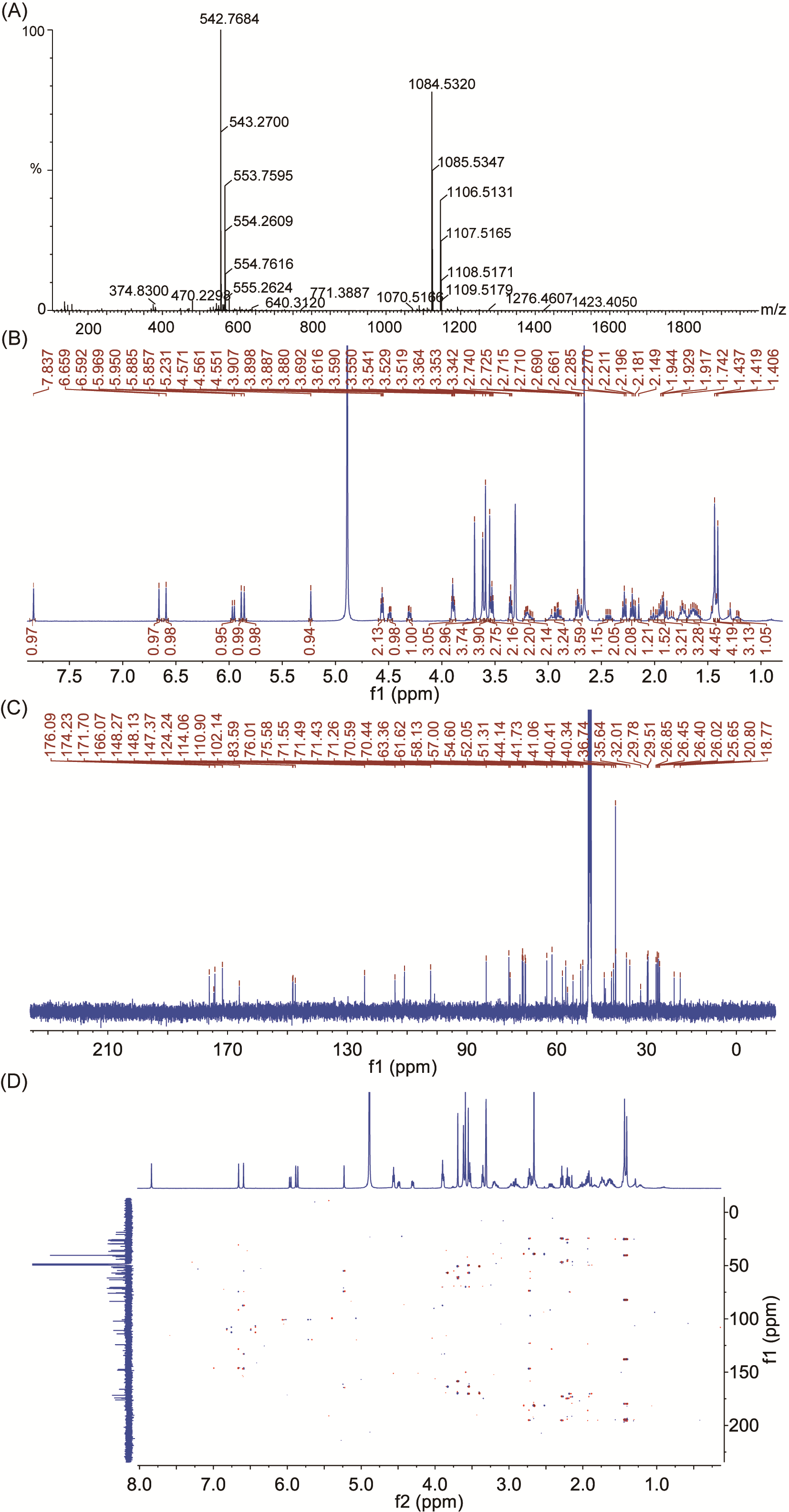


**Figure S2** **Structure identification of bio-HHT**. (A) HR-ESI-MS spectrum of bio-HHT. (B) ^1^H NMR spectrum of bio-HHT in CD_3_OD (400 MHz). (C) ^13^C NMR spectrum of bio-HHT in CD_3_OD (100 MHz). (D) HMBC spectrum of bio-HHT in CD_3_OD (400 MHz). Bio-HHT (7 mg, 98% purity) was obtained as a yellow oil after lyophilization. ^1^H NMR (400 MHz, CD3OD) 7.84 (s, 1H), 6.60 (s, 1H), 6.59 (s, 1H), 5.96 (d, 1H), 5.89 (s, 1H), 5.86 (s, 1H), 5.23 (s, 1H), 4.56 (t, 2H), 4.49 (dd, 2H), 3.89 (t, 2H), 3.88 (d, 1H), 3.69 (s, 3H), 3.62 (s, 4H), 3.59 (s, 4H), 3.54 (t, 2H), 3.52 (s, 3H), 3.35 (t, 2H), 1.44 (s, 3H), 1.41 (s, 3H), (+)-HRESIMS m/z 1084.5320 [M + H]^+^.


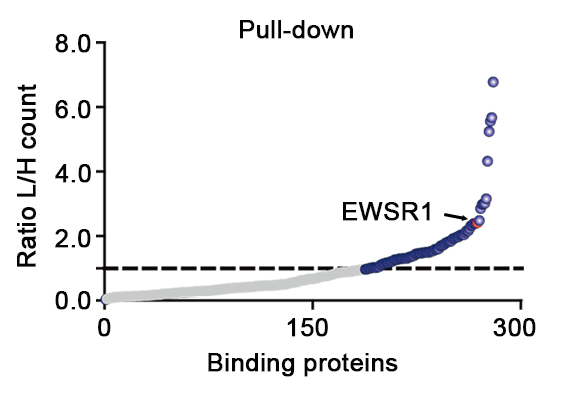


**Figure S3** Identification of HHT target proteins using pull-down technology combined with stable isotope labeling by amino acids in cell culture (SILAC). NB4 Cells are cultured in media containing isotopically labeled amino acids. Bio-HHT probe was utilized for pull-down assay. Target proteins are identified through mass spectrometry analysis based on the SILAC ratio (light/heavy).


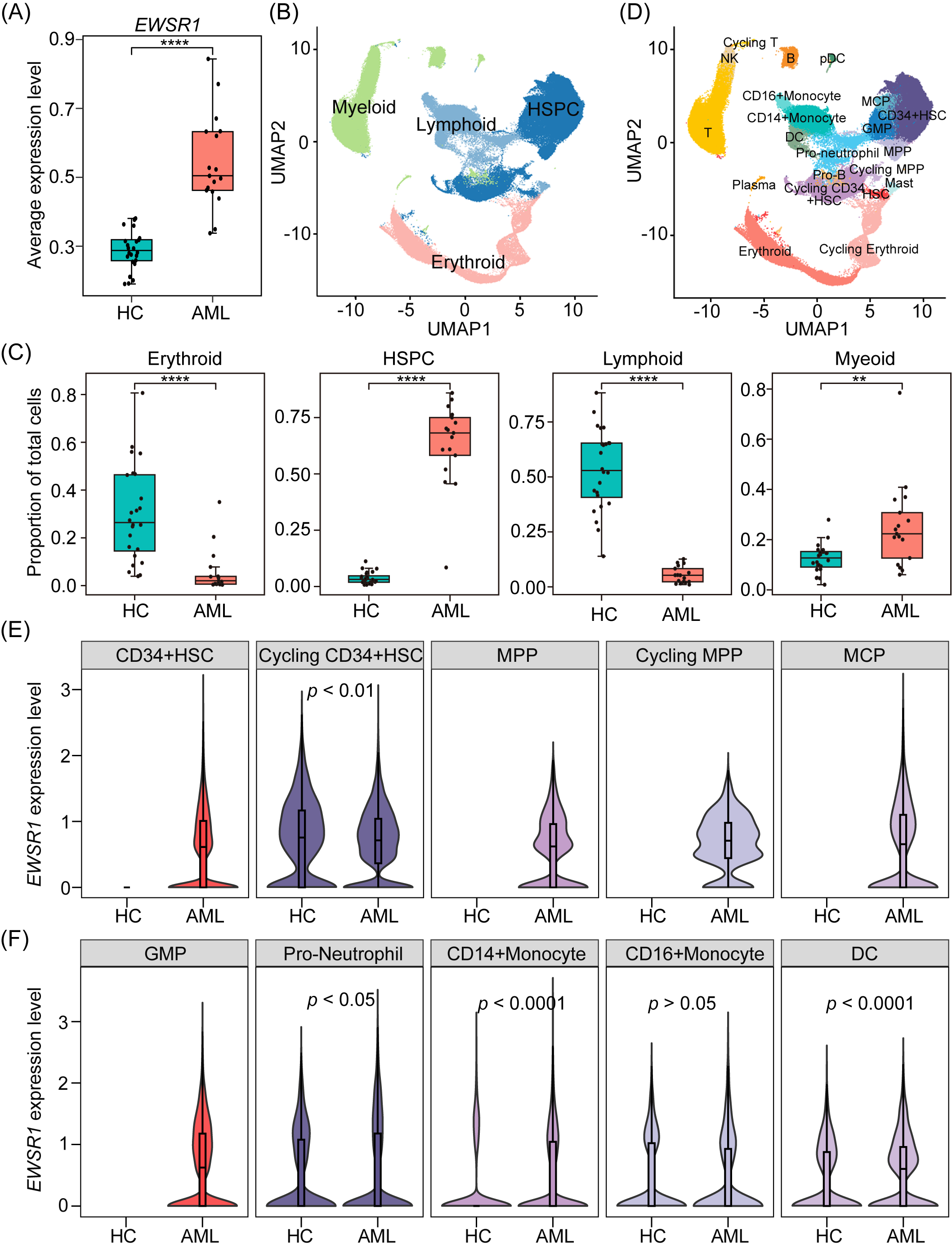


**Figure S4 Single-cell RNA sequencing reveals EWSR1 as a driver of AML progression.** (A) Evaluation of the average expression levels of EWSR1 gene in AML and HC cells (*****p* < 0.0001). (B) UMAP showing four major cell types, including hematopoietic stem progenitor cell (HSCP), myeloid, erythroid, and lymphoid. (C) Boxplot showing proportion distribution of four major cell types in normal and AML individuals. (D) UMAP showing 21 subpopulations in AML and HC samples. (E) Analysis of EWSR1 expression level in HSPC subsets, including CD34+ HSC, cycling CD34+ HSC, MPP, cycling MPP and MCP. (F) Analysis of EWSR1 expression level in myeloid subsets, including GMP, pro-neutrophil, CD14+ Monocyte, CD16+ Monocyte and DC. Data was presented as mean ± SD. Statistical analysis was performed using the two-tailed Wilcoxon rank-sum test for pairwise comparisons. Significance was indicated as follows: ***p* < 0.01, *****p* < 0.0001.


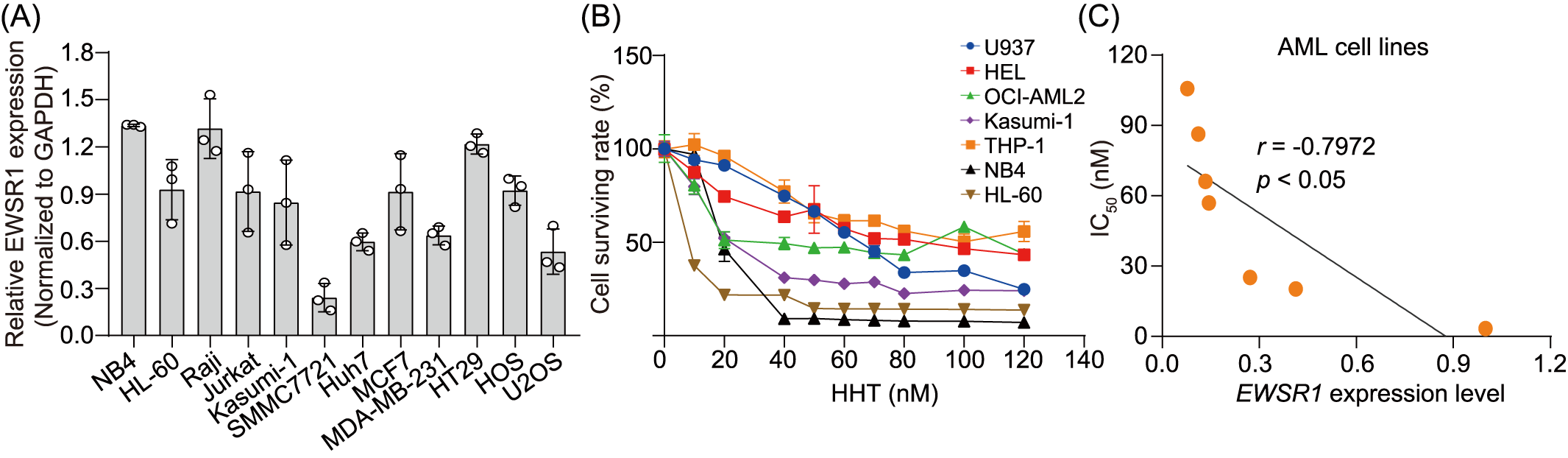


**Figure S5 EWSR1 expression and its association with HHT sensitivity in AML cells**. (A) The relative quantitative analysis of EWSR1 protein expression levels. Bar graph showing quantification of EWSR1 protein levels normalized to GAPDH control. Data are presented as mean ± SD (*n* = 3). (B) Cell viability assays showing the dose-dependent inhibitory effects of HHT on multiple AML cell lines, including U937, HEL, OCI-AML2, Kasumi-1, THP-1, NB4, and HL-60. Cells were treated with increasing concentrations of HHT for 48 h, and cell survival rates were measured by CCK-8 assay. Data are presented as mean ± SD from three independent experiments. (C) Correlation analysis between *EWSR1* expression levels and HHT sensitivity across AML cell lines.


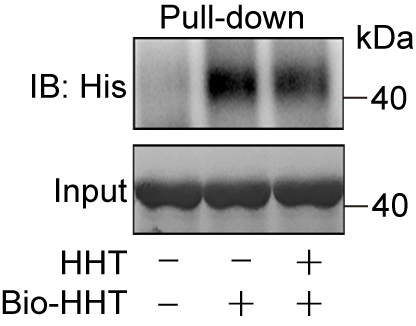


**Figure S6 The interaction between HHT and EWSR1-C recombinant protein was confirmed by pull-down assay in vitro**. The bio-HHT probe was conjugated to streptavidin magnetic beads, and purified EWSR1-C protein was used for the pull-down experiment.


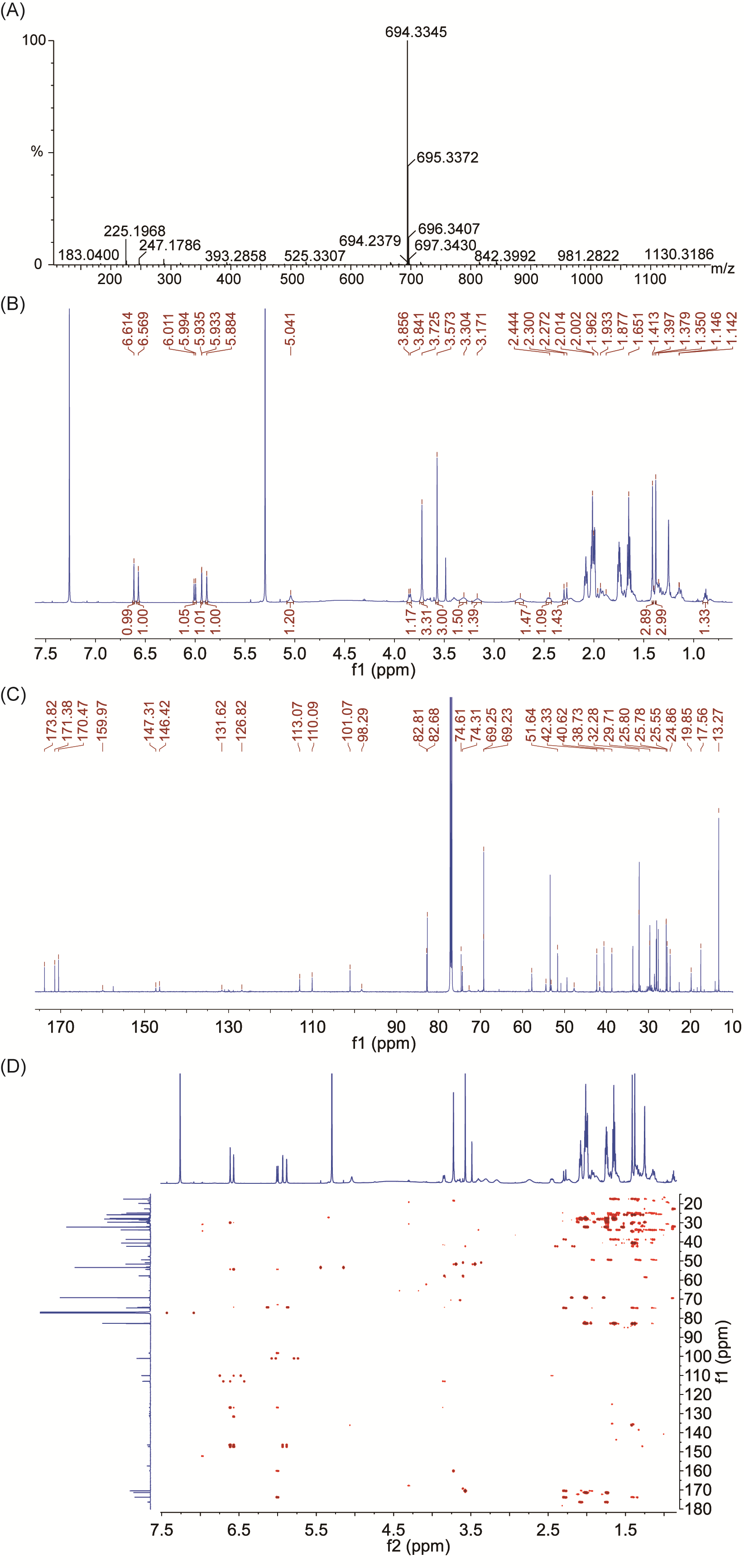


**Figure S7** **Structure identification of HHT-PAL probe.** (A) HR-ESI-MS spectrum of HHT-PAL probe. (B) ^1^H NMR spectrum of HHT-PAL probe in CDCl_3_ (500 MHz). (C) ^13^C NMR spectrum of HHT-PAL probe in CDCl_3_ (100 MHz). (D) HMBC spectrum of HHT-PAL in CDCl_3_ (500 MHz). HHT-PAL (10 mg, 99% purity) was obtained as yellow oil. ^1^H NMR (500 MHz, CDCl3), 6.61 (s, 1H), 6.57 (s, 1H), 6.00 (d, 1H), 5.93 (s, 1H), 5.88 (s, 1H), 5.04 (s, 1H), 3.85 (d, 1H), 3.73 (s, 1H), 3.57 (s, 3H), 3.30 (m, 1H), 3.17 (m, 1H), 2.74 (m, 4H), 2.44 (m, 1H), 2.29 (d, 1H), 2.24 (m, 1H), 2.01 (m, 1H), 2.00 (s, 1H), 1.99 (m, 1H), 1.93 (d, 1H), 1.88 (m, 2H), 1.70 (m, 1H), 1.67 (m, 1H), 1.65 (m, 3H), 1.62 (m, 1H), 1.61 (m, 2H), 1.41 (s, 3H), 1.40 (m, 1H), 1.38 (s, 3H), 1.36 (m, 1H), 1.35 (m, 3H), 1.15 (m, 1H). (+)-HRESIMS m/z 694.3345 [M + H]^+^.


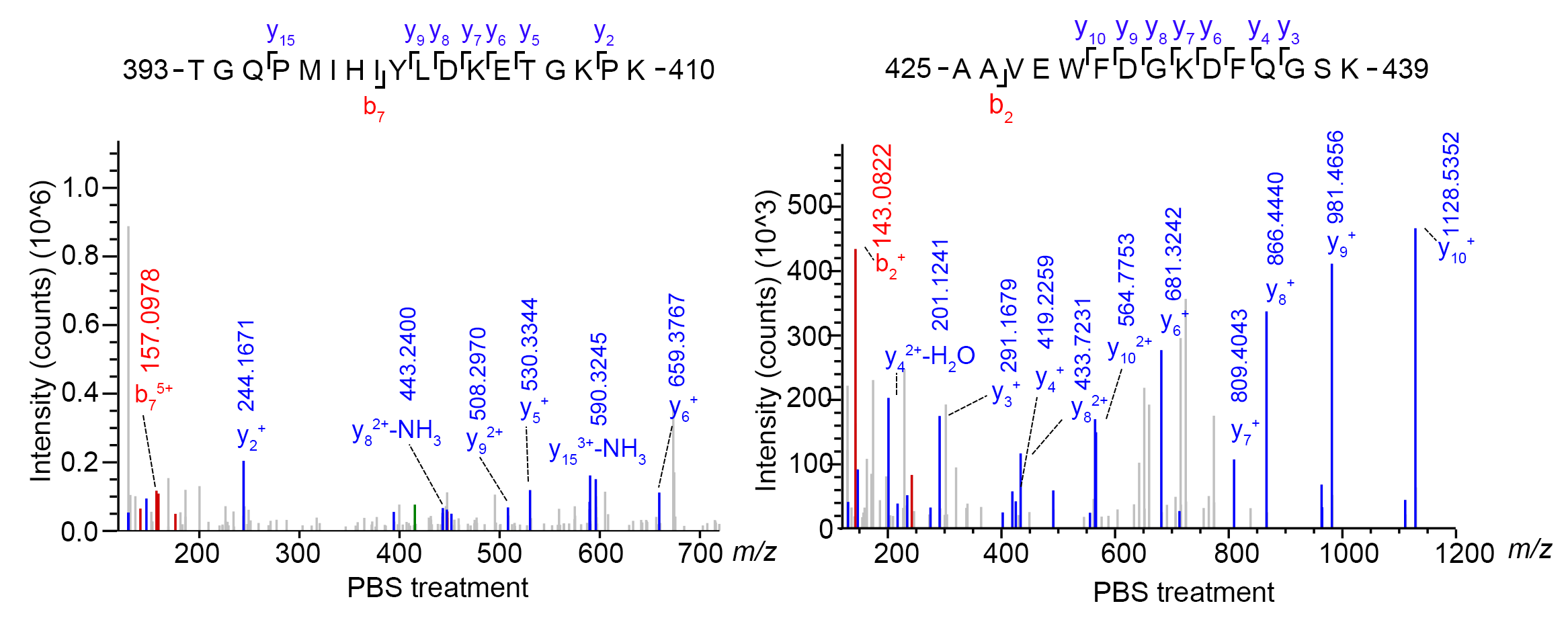


**Figure S8 LC-MS/MS analysis of covalently modified peptide of EWSR1-C**. Recombinant EWSR1-C protein was incubated with PBS overnight at 4 °C. Following incubation, the protein was subjected to trypsin digestion, and the modified peptides were analyzed by LC-MS/MS.


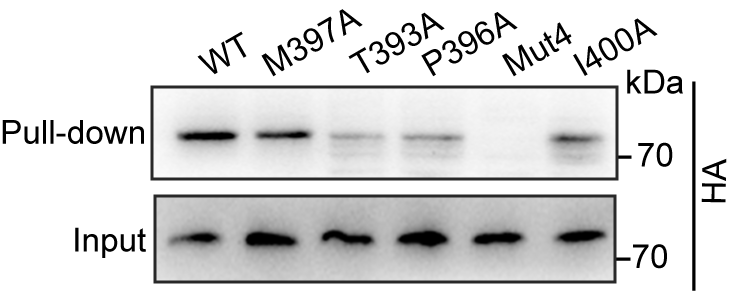


**Figure S9** **Pull-down assays validate critical residues in the EWSR1 RRM domain for interaction with HHT**. 293T cells were transfected with HA-tagged wild-type (WT) EWSR1 or mutant constructs (M397A, T393A, P396A, Mut4, I400A). Biotin-HHT was immobilized on streptavidin magnetic beads and incubated with cell lysates to capture interacting proteins. Bound proteins were eluted and analyzed by Western blotting using an anti-HA antibody.


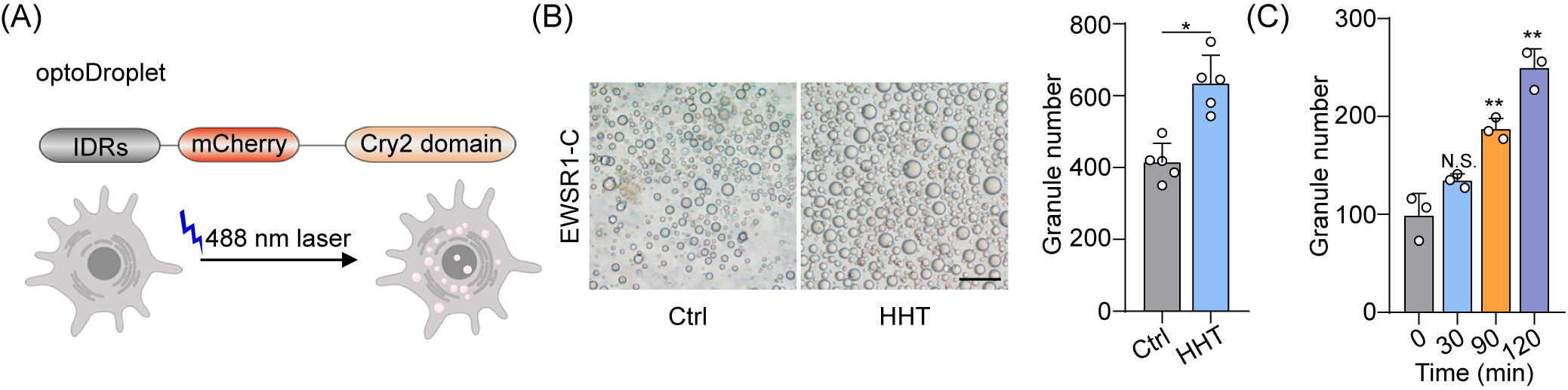


**Figure S10** **HHT modulates liquid-liquid phase separation of EWSR1**. (A) Principle of the optoDroplet experiment. The optoDroplet system utilizes light-induced protein-protein interactions to drive the phase separation of biomolecules into droplets. Upon exposure to 488 nm wavelengths of light, fusion proteins containing light-sensitive Cry2 domains undergo conformational changes, leading to the formation of liquid-liquid phase-separated droplets. (B) HHT influences the liquid-liquid phase separation (LLPS) of EWSR1-C *in vitro*. Data are presented as mean ± SEM; **p* < 0.05; *n* = 5. Scale bar: 100 μm. (C) Quantification of EWSR1-GFP puncta at each time point. Bars represent mean ± SD (***p* < 0.01; *N.S.* indicates no significance), *n* = 3.


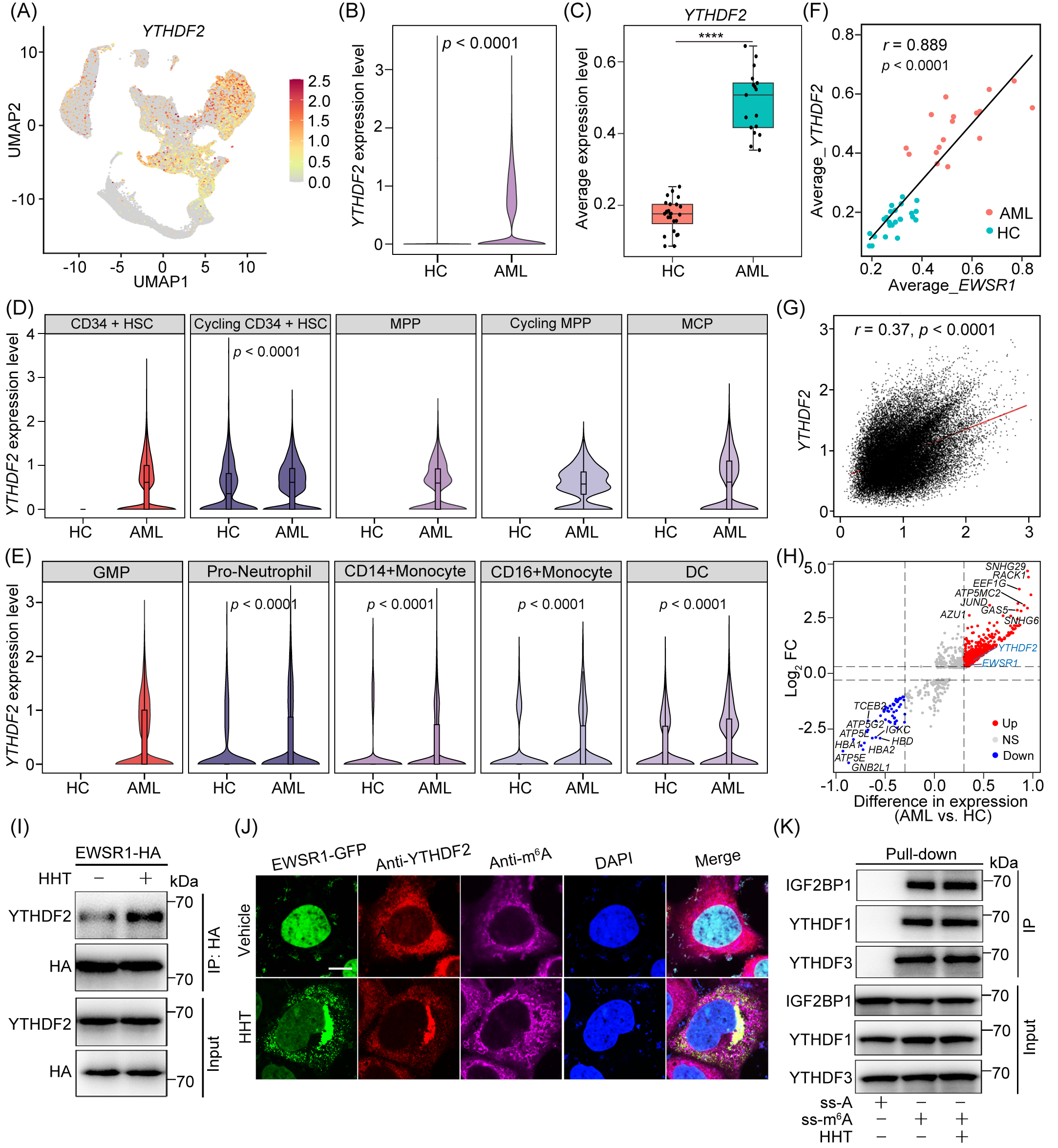


**Figure S11** ***YTHDF2* is highly expressed in AML**. (A) UMAP showing the expression of *YTHDF2* in the HC and AML cells. (B) Analysis of *YTHDF2* gene expression level in HC and AML individuals. (C) Evaluation of the average expression levels of *YTHDF2* gene expression level in HC and AML cells. (D) Examination of *YTHDF2* expression across HSPC subsets, including CD34+ HSC, cycling CD34+ HSC, MPP, cycling MPP, and MCP. (E) Evaluation of *YTHDF2* expression within myeloid subsets, such as GMP, pro-neutrophils, CD14+ monocytes, CD16+ monocytes, and DC. (F) Correlation analysis of *EWSR1* and *YTHDF2* gene expression levels in HC and AML samples, with each dot representing an individual sample. Pearson’s correlation coefficients are displayed. (G) Correlation analysis of gene expression levels for *EWSR1* and *YTHDF2* in HC and AML cells, where each dot corresponds to a single cell. Pearson's correlation coefficients are shown. (H) Scatter plot showing different expression genes between HC and AML. Red represents the upregulated genes. Blue represents the downregulated genes shared by both HC and AML cells. (I) The interaction between EWSR1 and YTHDF2 was assessed using a co-immunoprecipitation (CO-IP) assay. HEK293T cells were transfected with HA-tagged EWSR1 plasmid and the cell lysates were incubated with anti-HA magnetic beads. (J) Representative confocal images of cells expressing EWSR1-GFP (green), immunostained with anti-YTHDF2 (red), anti-m^6^A (purple). Nuclei were labeled with DAPI (blue). HEK293T cells were treated with HHT. Super-resolution microscopy was used to resolve subcellular distribution. Scale bar: 10 μm. (K) Biotinylated single-stranded RNA (ss-A) and m^6^A-modified RNA (ss-m^6^A) probes were used to pull down interacting proteins from cell lysates. The RNA-protein complexes were analyzed by western blot using antibodies against the m^6^A reader proteins IGF2BP1, YTHDF1, and YTHDF3. Data are presented as mean ± SD. Statistical significance was assessed using the two-tailed Wilcoxon rank-sum test for pairwise comparisons. Significance levels are indicated as ***p* < 0.0001.


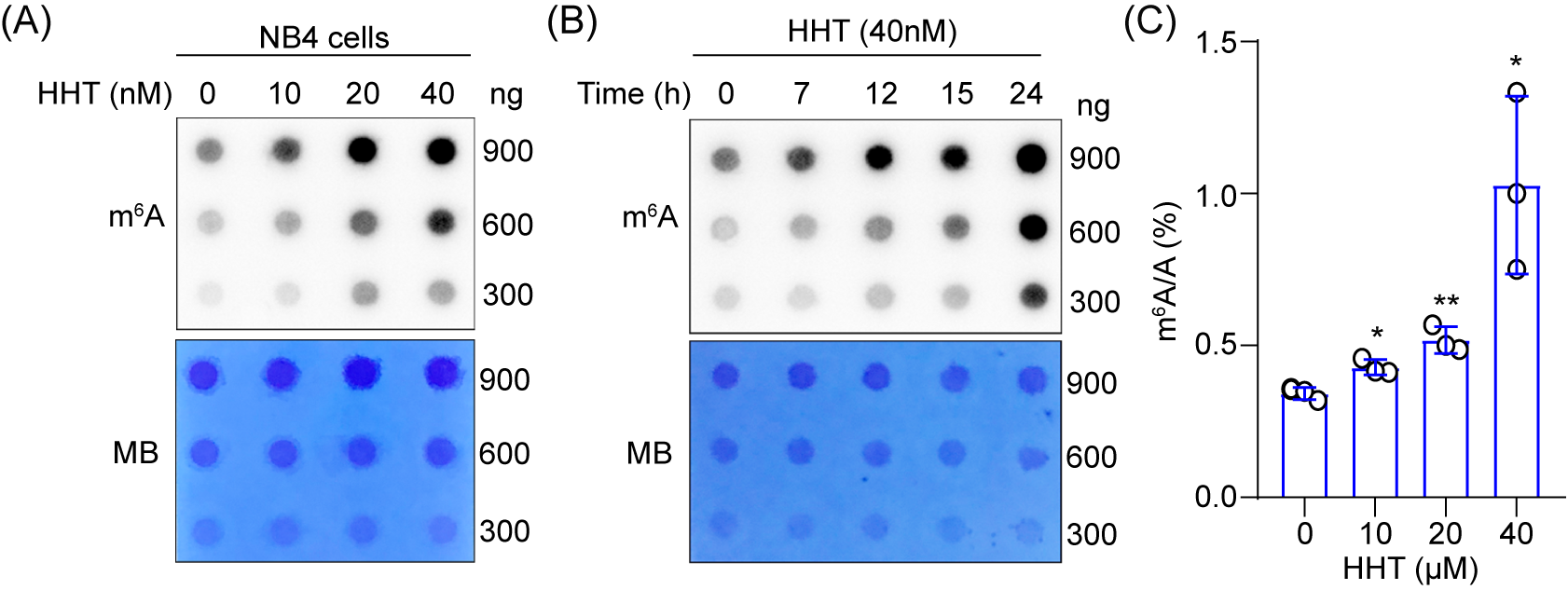


**Figure S12 HHT treatment increases m^6^A methylation levels in NB4 cells**. (A) The m^6^A dot blot assay in NB4 cells treated with different concentrations of HHT. MB, methylene blue staining (as loading control). (B) The m^6^A dot blot assay in NB4 cells treated with 40 nM HHT for different times. (C) The quantification of the m^6^A abundance within poly(A)+ RNA through LC-MS/MS analysis. Significance levels are annotated as: **p* < 0.05, ***p* < 0.01.


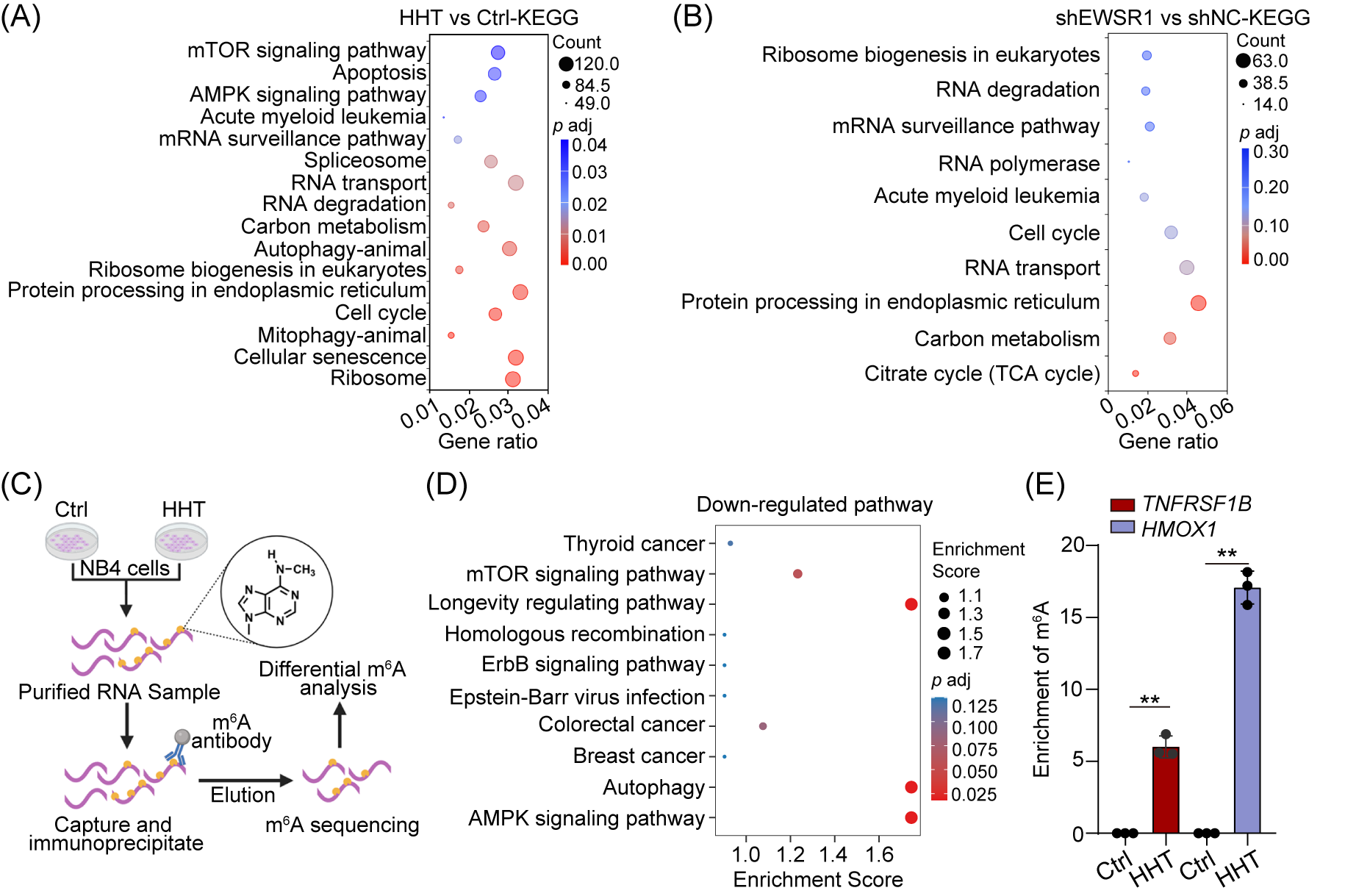


**Figure S13** **HHT targets EWSR1 to regulate m^6^A-modified pathways in leukemia cells**. (A) KEGG pathway enrichment analysis of differentially expressed genes in HHT-treated NB4 cells and shEWSR1 versus control NB4 cells. (B) KEGG pathway enrichment analysis of differentially expressed genes in shEWSR1 versus control NB4 cells. (C) Illustration of methylated RNA immunoprecipitation sequencing (MeRIP-seq) of NB4 cells treated with HHT. (D) KEGG pathway analysis of m^6^A-modified downregulated genes. (E) The m^6^A abundances in *TNFRSF1B* and *HMOX1* mRNA transcripts were detected by m^6^A-seq. Bars indicate mean ± SD, with individual biological replicates (*n* = 3 per group). Significance is indicated as ***p* < 0.01.
